# Non-dilemmatic social dynamics promote cooperation in multilayer networks

**Published:** 2026-01-01

**Authors:** Jnanajyoti Bhaumik, Naoki Masuda

**Affiliations:** 1 Department of Mathematics, State University of New York at Buffalo, NY 14260-2900, USA; 2 Gilbert S. Omenn Department of Computational Medicine and Bioinformatics, University of Michigan, MI 48109, USA; 3 Department of Mathematics, University of Michigan, MI 48109, USA; 4 Center for Computational Social Science, Kobe University, Kobe, 657-8501, Japan

## Abstract

Various theoretical and empirical studies have accounted for why humans cooperate in competitive environments. Although prior work has revealed that network structure and multiplex interactions can promote cooperation, most theory assumes that individuals play similar dilemma games in all social contexts. However, real-world agents may participate in a diversity of interactions, not all of which present dilemmas. We develop an evolutionary game model on multilayer networks in which one layer supports the prisoner’s dilemma game, while the other follows constant-selection dynamics, representing biased but non-dilemmatic competition, akin to opinion or fad spreading. Our theoretical analysis reveals that coupling a social dilemma layer to a non-dilemmatic constant-selection layer robustly enhances cooperation in many cases, across different multilayer networks, updating rules, and payoff schemes. These findings suggest that embedding individuals within diverse networked settings—even those unrelated to direct social dilemmas—can be a principled approach to engineering cooperation in socio-ecological and organizational systems.

## 1 Introduction

Altruistic behavior is a key feature of both humans and non-human organisms. Over past decades, studies of social dilemma games, most famously represented by the prisoner’s dilemma game, have revealed various mechanisms with which individuals cooperate when prosocial behavior is desirable for the society but apparently irrational for individuals. Such mechanisms include kin selection, repeated interactions between the same individuals, reputations, and population structure [1–3].

Networks, or population structure in general, promote prosocial behavior in multiple ways. For example, clustering of individuals, quantified by the abundance of short cycles composed of a few individuals, promotes cooperation because short cycles create locally connected clusters of cooperators that protect themselves against exploitation by non-cooperators [4–6]. Any edge (i.e., dyad) forming a network in fact creates assortative connectivity between cooperators, promoting cooperation [7]. Heterogeneity in the degree (i.e., the number of neighbors that an individual has) across individuals also promotes cooperation under some assumptions [8–10]. Time-varying nature of networks can also promote cooperation [11–14], whereas the opposite holds true in some situations [15]. All these network features are shared by a vast majority of empirical social networks [16–18].

In fact, humans, as well as animals and organizations, can interact with others in multiple social contexts and modalities. For example, we may interact with each other both in person and online. This situation can be modeled by a multilayer network, of which each network layer is defined by one type of edge (i.e., interaction) [19–22]. Various numerical studies have shown that cooperation can be enhanced in multilayer networks [23–27]. Su et al. pioneered a theoretical framework to understand cooperation in multilayer networks [28]. They assumed that each player is involved in a distinct prisoner’s dilemma game with peers in each network layer and that the payoff of each player that guides evolutionary dynamics in the different layers is the sum of the payoffs that the player obtains across all the network layers. They showed that multilayer networks enhance cooperation under broad conditions, compared to each network layer separately considered.

Social dilemmas are not the only situation that humans are coping with. Even if we face a social dilemma in our daily life, we would also be simultaneously involved in other types of social but non-dilemmatic interactions. A game formulation of this situation is to make individuals play different types of games in different network layers [27, 29–31]. Seminal studies also analyzed a related situation in which the social dilemma game and imitation of behavior occur in different network layers [32, 33]. How robust is enhanced cooperation in multilayer networks when individuals are not always participating in social dilemma games? Although there are some numerical results along this direction [27], can we provide theoretical underpinnings to elucidate how non-dilemmatic social dynamics modify the likelihood of prosocial behavior in dilemmatic situations? To answer these questions, we propose an evolutionary game model on two-layer networks (which can be readily extended to the case of more than two layers) in which players are involved in the prisoner’s dilemma game in one network layer and constant-selection dynamics in the other layer. The constant-selection dynamics is a simple social dynamics in which two types (e.g., opinions) compete in a population. It is equivalent to the biased voter model, a model of consensus formation dynamics used in mathematics [34, 35] and interdisciplinary physics [36, 37] for many years. By extending a recently developed theoretical framework [28], we show that the constant-selection dynamics can promote cooperation played in the opposite network layer in many cases. This study expands the set of scenarios that promote cooperation in multilayer populations and furnishes a theoretical framework to study agents’ multifaceted payoff-seeking behavior when social-dilemma interactions account for only part of their payoffs.

## 2 Model

**Figure 1:**
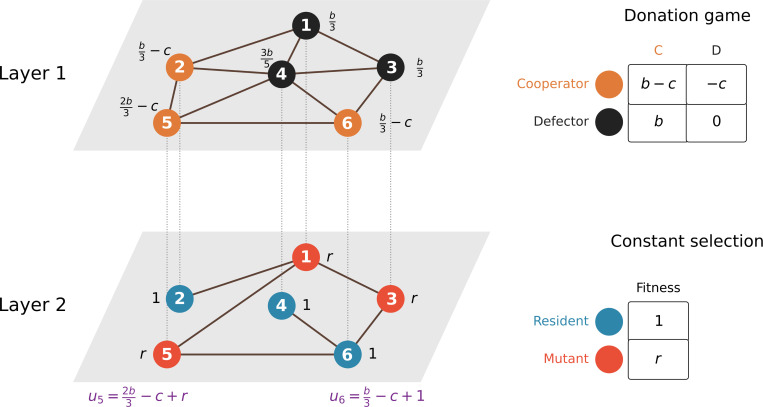
Schematic of the two-layer game. The donation game is played in layer 1. The constant-selection dynamics occurs in layer 2. The total payoff for each individual, which drives evolutionary dynamics in both layers, is the sum of the payoffs that the individual obtains from both layers. The figure shows the total payoffs for individuals 5 and 6 as examples. In both layers, replica nodes (shown by circles) that have a high total payoff tend to spread its type to its neighbors.

Figure 1 is a schematic of our evolutionary dynamics model on two-layer networks. We assume that there are N individuals. The replica node refers to a node of the undirected network layer. Therefore, there are 2N replica nodes, and each individual is represented by two replica nodes. Each edge directly connects two replica nodes in the same layer and represents one type of pairwise interaction between individuals. We assume that the individuals play the donation game, a special case of the prisoner’s dilemma game, with each of their neighbors in layer 1 and that the evolutionary dynamics are governed by constant selection in layer 2.

In layer 1, each ith individual is either a cooperator or a defector, encoded into $x_{i}=1$ and $x_{i}=0$, respectively. Each cooperator pays a cost c and provides a benefit b to each of its neighbors; this is called a pf goods scheme [38]; we will examine a different goods scheme later. The weight of an edge from the ith replica node to the jth replica node within layer L∈{1,2} is denoted by $w_{ij}^{\left[L\right]}$. We denote the weighted degree (which we simply call the degree in the following text) of the ith replica node in layer L by $s_{i}^{\left[L\right]}:=\sum_{j=1}^{N}w_{ij}^{\left[L\right]}$. The payoff of the ith replica node in layer 1 is given by

(1) $$u_{i}^{\left[1\right]}=-cx_{i}^{\left[1\right]}+\sum_{j=1}^{N}bp_{ij}x_{j}^{\left[1\right]},$$

where $p_{ij}=w_{ij}^{\left[1\right]}/s_{i}^{\left[1\right]}$. In layer 2, each node is assumed to be either the mutant or resident type. The fitness of the mutant and resident type is denoted by r and 1, respectively. Therefore, the payoff of the ith replica node in layer 2 is given by

(2) $$u_{i}^{\left[2\right]}=x_{i}^{\left[2\right]}(r-1)+1.$$

The total payoff of the ith individual, denoted by $u_{i}$, is assumed to be the sum of payoff across both layers, i.e., $u_{i}=u_{i}^{\left[1\right]}+u_{i}^{\left[2\right]}$.

We assume that the evolutionary dynamics is governed by the death-Birth (dB) updating rule. At the beginning of every time step in the evolutionary dynamics, we choose a replica node i in layer 1 uniformly at random (with probability 1/N) to update its strategy. Then, the neighbors of i in layer 1 compete to disseminate its strategy to i. A neighbor of i is chosen with the probability proportional to their fecundity, where fecundity of an individual i is defined as $F_{i}=1+\delta u_{i}$; note that δ(≥0) is the strength of selection. The type of j (i.e., cooperation or defection) replaces that of i in layer 1. Simultaneously, in layer 2, in a similar fashion, an individual j is selected for death uniformly at random. Next, a neighbor of j in layer 2 is selected for reproduction with the probability proportional to $u_{j}$. Then, the type of j (i.e., mutant or resident) replaces that of i in layer 2, concluding one time step of the evolutionary dynamics. This process is repeated until there are either only cooperators or only defectors in layer 1 and there are either only mutants or only residents in layer 2. Throughout the text, we assume weak selection, that is, δ≪1.

We use the dB updating rule for layer 1 because it is often used for studying prosociality on networks [7, 28, 39–42]. We use the same updating rule in layer 2 for simplicity. We call the combined updating rule the dB-dB rule. In fact, a different updating rule called the Birth-death (Bd) is more commonly used for constant-selection evolutionary dynamics [43, 44]. Therefore, we also investigate the Bd rule for layer 2. In this case, we call the combined updating rule the dB-Bd rule.

## 3 Results

### 3.1 Conditions under which cooperators and mutants are favored

We derived the condition under which the cooperator is favored in layer 1 and that under which the mutant is favored in layer 2 for arbitrary two-layer networks (see section S1). More specifically, we derived the fixation probability for a single cooperator introduced to the population of defectors in layer 1 under weak selection, and similar for a single mutant introduced in layer 2. The derivation expands that in [28], where individuals are assumed to play the donation game in both networks. A key observation that we have exploited in our derivation is that constant-selection dynamics can be expressed as evolutionary game dynamics where the payoff matrix entries do not depend on the opponent’s action. Nonetheless, the constant selection produces terms that are not present in the case of the two-layer donation game. After obtaining a general solution (i.e., last equation in section S1), we substitute the quantities for a given updating rule to obtain the conditions for the dB-dB and dB-Bd rules (see sections S2 and S3, respectively).

Under the dB-dB rule, we have found that the condition under which cooperation is favored in layer 1 is given by

(3) $$c\theta_{2}^{\xi [1]}+b\left(\theta_{1}^{\xi [1]}-\theta_{3}^{\xi [1]}\right)-(r-1)\phi_{2,0}^{\xi [1,2]}>0.$$

Here, $\theta_{n}^{\xi [1]}$ is a recursively calculated quantity depending on the n-step transition probability matrix of the random walk on the layer-1 network and its stationary probability at each replica node. Quantity $\phi_{n,m}^{\xi [1,2]}$ is similarly obtained, but the random walk depends on both layers: an n-step random walk in layer 1, followed by an m-step random walk in layer 2, and also the states of the replica nodes in layer 1 before the walk and the states of the replica nodes in layer 2 after the walk.

Under the same dB-dB rule, the condition under which the mutant is favored in layer 2 is given by

(4) $$-(r-1)\theta_{2}^{\xi [1]}+c\phi_{2,0}^{\xi [1,2]}+b\left(\phi_{0,1}^{\xi [1,2]}-\phi_{2,1}^{\xi [1,2]}\right)>0.$$

### 3.2 Classification of two-layer networks and exhaustive examination of networks with N=6 individuals

#### 3.2.1 Evolution of cooperators

Let us more closely examine the conditions given by Eqs. (3) and (4). When r=1, Eq. (3) reduces to the condition for favoring cooperation in one-layer networks [28, 45]. This is because setting r=1 implies that the payoff from the constant-selection layer (i.e., layer 2) is equal to 1 for all individuals and therefore only makes the selection weaker for the donation game layer (i.e., layer 1).

To understand general cases, we first assume that $\theta_{1}^{\xi [1]}-\theta_{3}^{\xi [1]}>0$ and rewrite Eq. (3) as follows:

(5) $$\frac{b}{c}>{\left(\frac{b}{c}\right)}^{*}\equiv \frac{-\theta_{2}^{\xi [1]}}{\theta_{1}^{\xi [1]}-\theta_{3}^{\xi [1]}}+\frac{(r-1)\phi_{2,0}^{\xi [1,2]}}{c\left(\theta_{1}^{\xi [1]}-\theta_{3}^{\xi [1]}\right)}.$$

**Figure 2:**
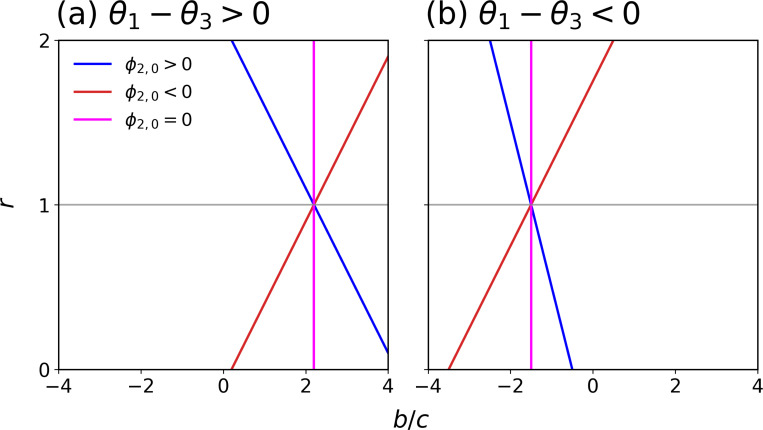
${\left(b/c\right)}^{*}$ for various values of r. (a) $\theta_{1}-\theta_{3}>0$. (b) $\theta_{1}-\theta_{3}<0$. In (a), cooperation is favored when b/c is larger than the value specified by the line, whose slope depends on $\phi_{2,0}$. In (b), spite is favored when b/c is smaller than the value specified by the line depending on the $\phi_{2,0}$ value.

When r=1, it is known that ${\left(b/c\right)}^{*}$ is positive because $\theta_{2}^{\xi [1]}<0$ [13,28,45], and Eq. (5) gives the condition for cooperation. Relative to this one-layer baseline case first shown in [45], coupling with the constant-selection dynamics layer, i.e., r≠1, changes ${\left(b/c\right)}^{*}$ as follows. Note that (r−1)/c is the relative weight of the constant selection to the donation game. We distinguish between the following three cases (see Fig. 2(a)).

1. If $\phi_{2,0}^{\xi [1,2]}>0$, then ${\left(b/c\right)}^{*}$ is lower in the two-layer than the one-layer network if 0≤r<1, with the two-layer network easing cooperation. In contrast, the two-layer network makes cooperation harder if r>1.
2. If $\phi_{2,0}^{\xi [1,2]}<0$, then ${\left(b/c\right)}^{*}$ is lower in the two-layer than the one-layer network if $1<r<r^{*}$, facilitating the cooperation. The converse holds true for 0<r<1.
3. If $\phi_{2,0}^{\xi [1,2]}=0$, then ${\left(b/c\right)}^{*}$ does not depend on r such that ${\left(b/c\right)}^{*}$ remains the same as the case of r=1.

In case 1, if $\phi_{2,0}^{\xi [1,2]}>-c\theta_{2}^{\xi [1]}(>0)$ and $0<r<r^{*}\equiv 1+\left(c\theta_{2}^{\xi [1]}/\phi_{2,0}^{\xi [1,2]}\right)(<1)$, then cooperation is favored when $b/c>{\left(b/c\right)}^{*}$ with a negative threshold ${\left(b/c\right)}^{*}$. It should be noted that, when the spite behavior is favored, the condition generally reads $b/c<{\left(b/c\right)}^{*}<0$ [45], while the inequality is in the opposite direction in the present case. In this case, the interpretation of the result is difficult. Note that the same behavior can occur when players are involved in different donation games in each network layer [28]. However, we confirmed in our numerical simulations described in the following text that this case never occurs to the best of our effort. Similarly, in case 2, if $r>r^{*}(>1)$, cooperation is favored when $b/c>{\left(b/c\right)}^{*}$ with a negative ${\left(b/c\right)}^{*}$. However, we have confirmed that this situation occurs only when r is much larger than 1, where the weak-selection assumption is compromised.

Second, when $\theta_{1}^{\xi [1]}-\theta_{3}^{\xi [1]}<0$, we obtain Eq. (5) but with the inequality being flipped. Then, the first term on the right-hand side of Eq. (5) is negative. Therefore, if r=1 or $\phi_{2,0}^{\xi [1,2]}=0$ (see Fig. 2(b)), then the condition reads $b/c<{\left(b/c\right)}^{*}<0$ such that spite is favored with the same value of ${\left(b/c\right)}^{*}$ as that for the one-layer network. If $\phi_{2,0}^{\xi [1,2]}\neq 0$, depending on whether $\phi_{2,0}^{\xi [1,2]}$ is positive or negative, spite occurs more easily or less easily for different ranges of of r, relative to r=1 (see Fig. 2(b)). Interpretation of the condition for the spite behavior $b/c<{\left(b/c\right)}^{*}$ would be difficult if ${\left(b/c\right)}^{*}$ is positive. However, we again numerically confirmed that this unphysical behavior only occurs when r is much larger than 1.

Finally, when $\theta_{1}^{\xi [1]}-\theta_{3}^{\xi [1]}=0$, cooperation is favored if $\theta_{2}^{\xi [1]}>\frac{(r-1)\phi_{2,0}^{\xi [1,2]}}{c}$.

In this manner, we can classify evolutionary outcomes with a linear analysis assuming weak selection. To assess which outcomes are frequent, we exhaustively considered unique two-layer networks with six individuals and all possible unique initial conditions with just one cooperator in layer 1 and one mutant in layer 2. Following the convention, we use the same initial condition (i.e., single cooperator and single mutant) in all the following numerical simulations as well. We set c=1 in this and the following numerical simulations unless we state otherwise. We show the classification of the 2,763,739 unique pairs of two-layer network and initial condition in the left panel of Table 1. We have several observations: First, more than 80% of the unique pairs of network and initial condition yield negative $\theta_{1}^{\xi [1]}-\theta_{3}^{\xi [1]}$, implying spite behavior. In fact, this result is not specific to two-layer networks because setting r=1 provides the condition for one-layer networks and the sign of $\theta_{1}^{\xi [1]}-\theta_{3}^{\xi [1]}$ is independent of r. Second, the condition for cooperation when $\theta_{1}^{\xi [1]}-\theta_{3}^{\xi [1]}=0$ is hard to interpret, but this case rarely occurs. Third, by additional numerical simulations, we confirmed that there is no case in which cooperation is selected for $b/c>{\left(b/c\right)}^{*}$ with a negative ${\left(b/c\right)}^{*}$ or the spite is selected for $b/c<{\left(b/c\right)}^{*}$ for a positive ${\left(b/c\right)}^{*}$ when r is sufficiently close to 0. The result that less than 20% networks with N=6 individuals foster cooperation is underwhelming. However, we will later see that cooperation rather than spite is favored in a majority of larger networks.

#### 3.2.2 Evolution of mutants

We turn to the condition under which the mutant is favored in the two-layer network, Eq. (4). Because $\theta_{2}^{\xi [1]}<0$, we can rewrite Eq. (4) as

(6) $$r>r^{*}\equiv 1+\frac{b\left(\phi_{0,1}^{\xi [1,2]}-\phi_{2,1}^{\xi [1,2]}\right)+c\phi_{2,0}^{\xi [1,2]}}{\theta_{2}^{\xi [1]}}\equiv 1+A.$$

This result is in stark contrast with that for one-layer networks because, in two-layer networks, both the payoff of the donation game and the structure of the two-layer networks in both layers influence the propensity that the mutant is favored through A. Remarkably, mutants whose fitness (i.e., r) is smaller than that of the resident type (i.e., 1) can be favored if A<0. If A>0, then r has to be sufficiently larger than 1 for the mutant to be favored. Another observation is that setting b/c to the ${\left(b/c\right)}^{*}$ value for the one-layer network does not lead to $r^{*}=1$ in general. Therefore, in contrast to the condition for cooperation, for which setting r=1 recovered the one-layer network results, we do not have a mathematically solid baseline one-layer case for the constant selection layer. Furthermore, Eq. (6) indicates that b and c independently affect $r^{*}$, and whether an increase in b or c increases or decreases $r^{*}$ depends on the network structure.

**Figure 3:**
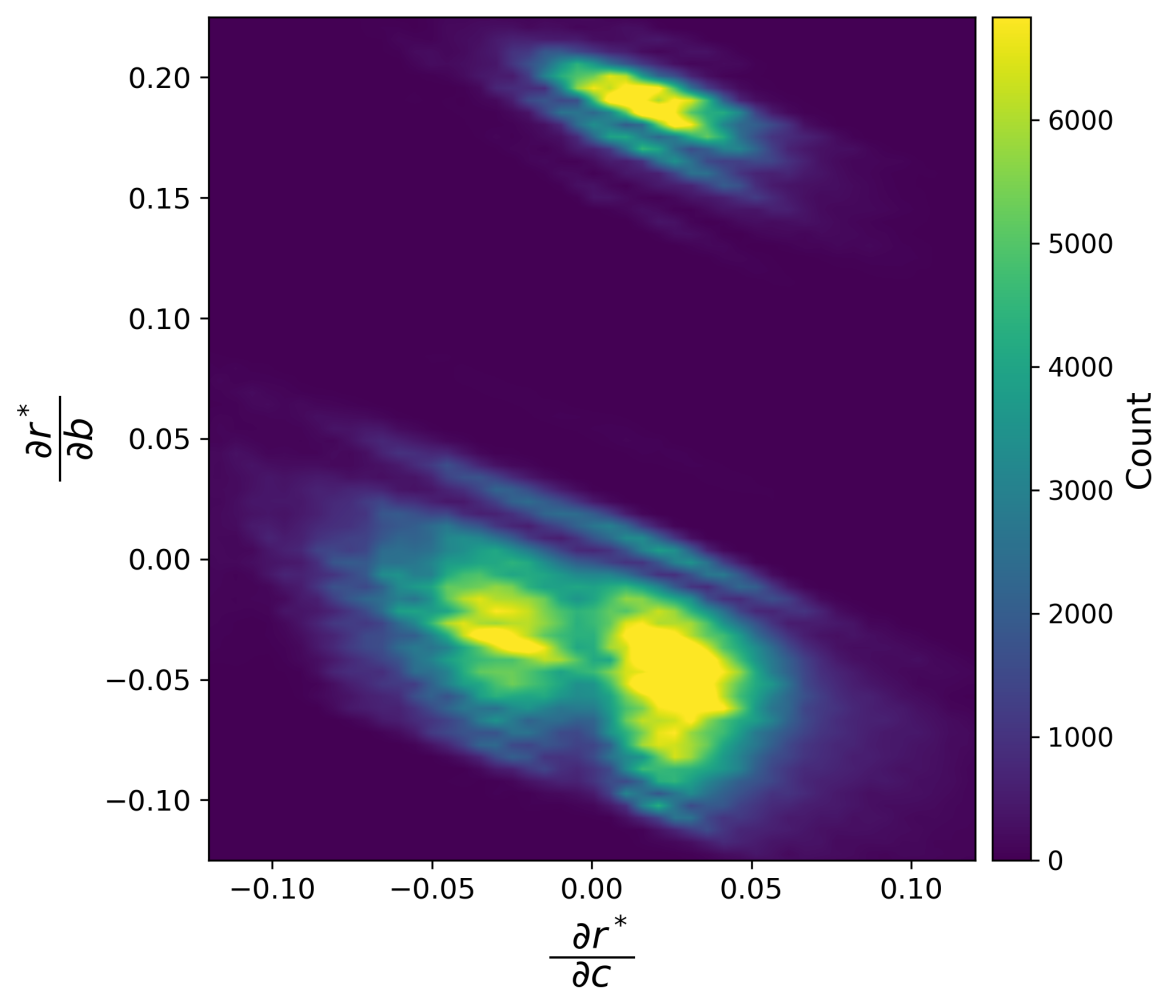
Distribution of $dr^{*}/db$ and $dr^{*}/dc$ over the 2,763,739 unique pairs of the two-layer network with six individuals and initial condition. This is a non-smoothed two-dimensional histogram.

To examine responsiveness of $r^{*}$ to the coupling with the donation game network layer, we investigated the same 2,763,739 unique pairs of two-layer network with N=6 individuals and initial condition to calculate $dr^{*}/db=\left(\phi_{0,1}^{\xi [1,2]}-\phi_{2,1}^{\xi [1,2]}\right)/\theta_{2}^{\xi [1]}$ and $dr^{*}/dc=\phi_{2,0}^{\xi [1,2]}/\theta_{2}^{\xi [1]}$. We show the distribution of $\left(dr^{*}/db\right.,\left.dr^{*}/dc\right)$ in Fig. 3. The figure suggsts that $dr^{*}/db$ and $dr^{*}/dc$ can have either sign depending on the network and initial condition. The effect of b and c on $r^{*}$ is modest; the average of $\left|dr^{*}/db\right|$ and $\left|dr^{*}/dc\right|$ over all the possible networks and initial condition is 0.0351 and 0.0665, respectively (with ranges $-0.211\leq \left|dr^{*}/db\right|\leq 0.294$ and $-0.262\leq \left|dr^{*}/dc\right|\leq 0.247$; the density of unique pairs of two-layer network and initial condition is invisibly small outside the $\left(dr^{*}/db,dr^{*}/dc\right)$ region shown in the figure). The different stripes present in Fig. 3 partially owe to the network structure and initial condition of layer 1 (see section S4). We conclude that the b and c values modestly, but consistently, affects $r^{*}$ in almost all cases.

#### 3.3 Sample two-layer networks

**Figure 4:**
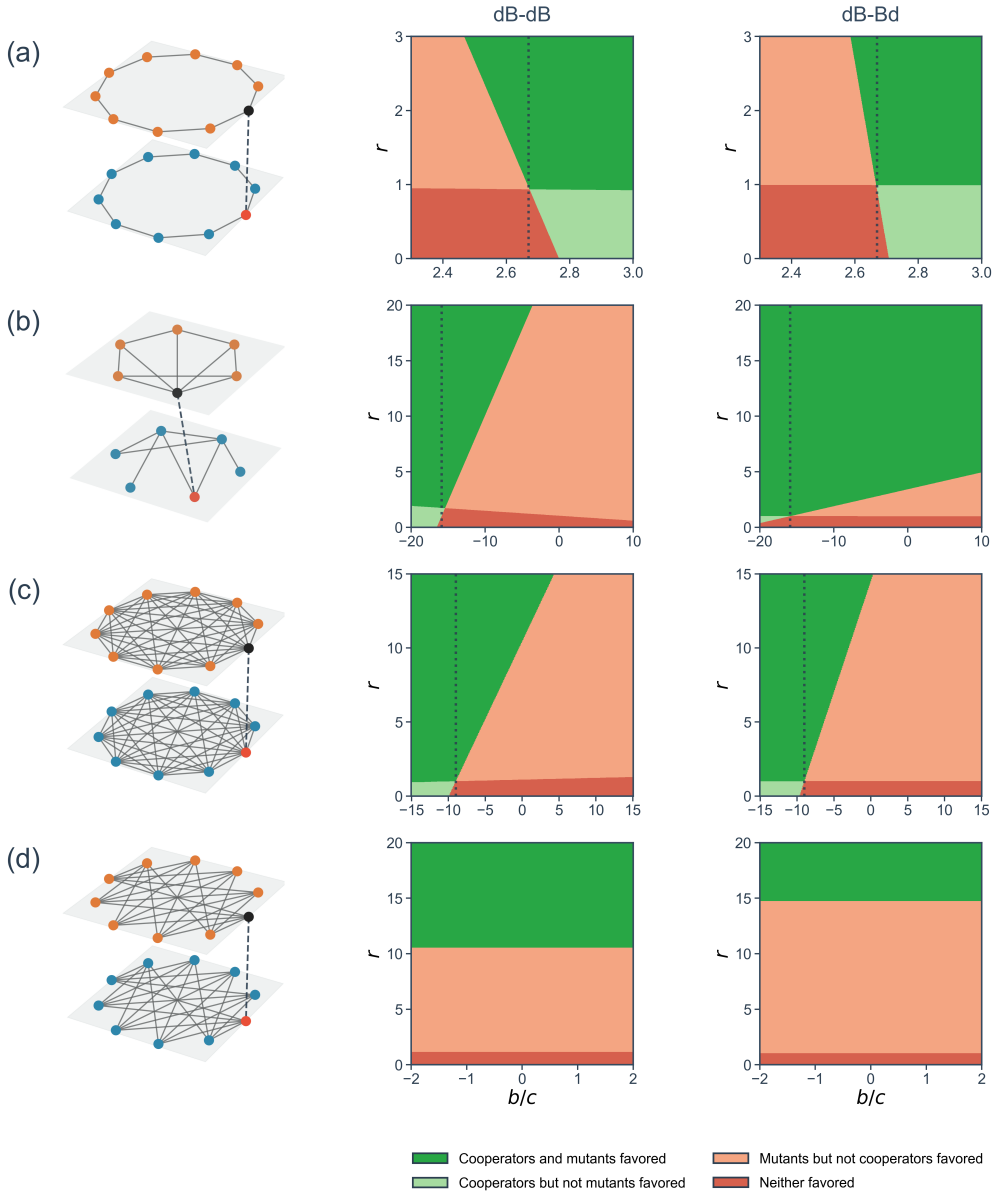
Selection of cooperators and mutants in four two-layer networks. Each row corresponds to the two-layer network and its initial condition visualized in the left panel. The colors of the replica nodes are the same as those used in Fig. 1; orange, black, blue, and red represent cooperator, defector, resident, and mutant, respectively. The second column of each row of the figure shows the results under the dB-dB updating rule. The third column shows the results under the dB-Bd updating rule. Each colored region shows the (b/c,r) region in which selection favors or disfavors cooperators in layer 1 or mutants in layer 2. (a) Coupled ring networks with N=10 individuals. We obtain ${\left(b/c\right)}^{*}=8/3$ for the uncoupled one-layer ring, shown by the dotted lines. (b) Coupled heterogenous networks with N=6. We obtain ${\left(b/c\right)}^{*}=-15.89$ for the uncoupled layer-1 network. (c) Coupled complete graphs with N=10. The one-layer counterpart yields ${\left(b/c\right)}^{*}=-9$. (d) Coupled complete bipartite graphs with N=10. The one-layer counterpart yields ${\left(b/c\right)}^{*}=\infty$.

To examine whether various two-layer networks favor cooperators or mutants and how, we examine larger model networks. The first two of them were used in [28]. First, consider a coupled ring network with N=10 individuals shown in Fig. 4(a). When each layer evolves independently, one obtains ${\left(b/c\right)}^{*}=8/3$. When the two layers are coupled, r>1 causes cooperation to emerge in layer 1 under a more generous condition than in the case of a one-layer network, i.e., ${\left(b/c\right)}^{*}<8/3$ (see the middle panel in Fig. 4(a)). For example, we obtain ${\left(b/c\right)}^{*}=2.57$ when r=2. Mathematically, in the limit of infinitely large two-layer ring network, ${\left(b/c\right)}^{*}$ decreases by 4(r−1)/c from the one-layer case (i.e., ${\left(b/c\right)}^{*}=1/2$ if the individual that initially cooperates in layer 1 is of the mutant type in layer 2 (see section S5). In other cases, the effect of the constant-selection layer to modulate ${\left(b/c\right)}^{*}$ diminishes as the size of the ring increases.

Second, we investigated a heterogenous two-layer network shown in Fig. 4(b). In this network, each layer promotes the evolution of spite (i.e., ${\left(b/c\right)}^{*}<0$) when the two layers are uncoupled. We find for this two-layer network that spite is favored with a smaller punishment (i.e., negative b/c values closer to 0) when r>1 relative to the one-layer network and vice versa when r<1.

Third, we investigated a two-layer network composed of two complete graph layers shown in Fig. 4(c). The results are qualitatively the same as those for the heterogeneous two-layer network investigated in Fig. 4(b). For this coupled complete graph, we analytically obtained the condition for favoring spite as $b/c<{\left(b/c\right)}^{*}=-(N-1)-\frac{r-1}{c}\cdot \frac{2{\left(N-1\right)}^{2}}{N(2N-3)}$; see section S6 for the derivation. This result implies that spite can occur only in small coupled complete graphs because ${\left(b/c\right)}^{*}\to -\infty$ as N→∞.

Fourth, we investigated a two-layer network composed of two complete bipartite networks shown in Fig. 4(d). We find that selection favors cooperation if r is approximately larger than 10.5, regardless of the value of b/c (see the middle panel of Fig. 4(d)). In contrast, a single complete bipartite network gives ${\left(b/c\right)}^{*}=\infty$, i.e., no cooperation.

Fifth, we also derived analytical results for the coupled star graph of arbitrary size (section S7).

These results are similar to those when the individuals play different donation games in two network layers of similar sizes [28]. Regardless of whether the second layer is subject to the social dilemma game or constant-selection dynamics, coupling of networks modulates the threshold value of b/c, i.e., ${\left(b/c\right)}^{*}$, for cooperation or spite. Quantitatively, in the coupled ring network (Fig. 4(a)), ${\left(b/c\right)}^{*}$ decreases from 8/3 in the case of the one-layer ring network to 1.74 when the donation game with b/c=10 is played on the other ring layer [28]. In our model, reduction of ${\left(b/c\right)}^{*}$ to 1.74 requires r=10.78, which is by chance close to the aforementioned value of b/c=10.

### 3.4 Random graphs

As a next test, we examined two types of larger random two-layer network models. The first model is a two-layer Erdős-Rényi (ER) random graph. For each layer, we generate an instance of the ER random graph with N=15 replica nodes with the probability of edge between each pair of nodes $p_{L}\in \{0.1,0.2,0.3,0.4,0.5\}$ for layer L∈{1,2}. The two network layers are independently generated. We evaluated all the 225 possible initial conditions with one cooperator in layer 1 and one mutant in layer 2 for each generated two-layer network with given $p_{1}$ and $p_{2}$.

**Figure 5:**
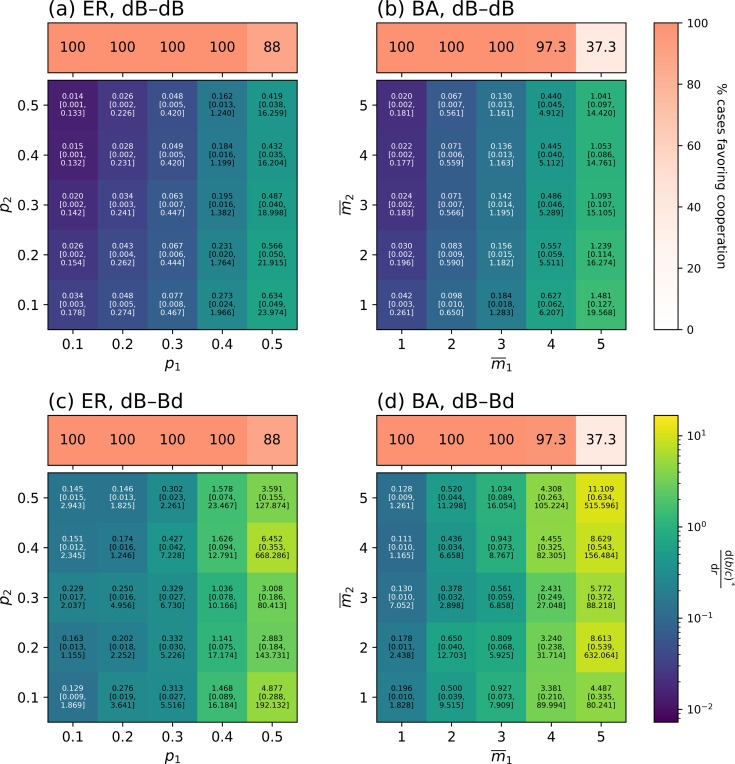
Evolution of cooperation in coupled ER and BA networks. (a) ER, dB-dB rule. (b) BA, dB-dB rule. (c) ER, dB-Bd rule. (d) BA, dB-Bd rule. The upper part of each panel shows the fraction of pairs of one-layer network and its initial condition that yield cooperation when $b/c>{\left(b/c\right)}^{*}$ with a threshold value ${\left(b/c\right)}^{*}>0$. The fraction values are the same between (a) and (c) and between (b) and (d) because this fraction only depends on the layer-1 network. The lower part of each panel shows the median along with the 5th and 95th percentiles (in square brackets) of $\left|\text{d}{\left(b/c\right)}^{*}/\text{d}r\right|$ for pairs of two-layer network and initial condition. Each two-layer network is composed of N=15 individuals.

We first counted the fraction of the layer-1 ER random graphs for which cooperation as opposed to spite is favored when $b/c>{\left(b/c\right)}^{*}$ with a positive value of ${\left(b/c\right)}^{*}$. We are interested in this case because, if this is the case, it is likely that one can choose a value of r that lowers ${\left(b/c\right)}^{*}$ in the two-layer network such that cooperation is facilitated by the coupling with the constant-selection network layer (i.e., layer 2). The upper part of Fig. 5(a) shows the fraction of pairs of one-layer ER random graph and initial condition yielding ${\left(b/c\right)}^{*}>0$. Because this inquiry only concerns one-layer networks, we only show the result as a function of $p_{1}$. We find that the all instances of networks enable cooperation when $p_{1}\leq 0.4$ and that 88% of them do so when $p_{1}=0.5$. These values are much larger than for smaller networks; compare these numbers with those for networks with N=6 individuals shown in Table 1.

Next, we examined, among the runs yielding cooperation in layer 1, how much ${\left(b/c\right)}^{*}$ can be changed by the constant-selection dynamics. To quantify this effect, we measured $\left|\text{d}{\left(b/c\right)}^{*}/\text{d}r\right|$, i.e., how much ${\left(b/c\right)}^{*}$ moves by changing r for each pair of two-layer network and initial condition. For each pair of $p_{1}$ and $p_{2}$, we show in the lower part of Fig. 5(a) the median along with the 5th and 95th percentiles of $\left|\text{d}{\left(b/c\right)}^{*}/\text{d}r\right|$. We show the median and percentiles instead of the mean and standard deviation because there are occasional outliers that yield a huge $\left|\text{d}{\left(b/c\right)}^{*}/\text{d}r\right|$ relative to typical pairs of two-layer network and initial condition. However, we have confirmed that the tendency that we are reporting with the median and percentiles remains similar when we measure the mean and standard deviation of $\left|\text{d}{\left(b/c\right)}^{*}/\text{d}r\right|$ (see section S8). In Fig. 5(a), we find that $\left|\text{d}{\left(b/c\right)}^{*}/\text{d}r\right|$ is larger for denser layer-1 networks (i.e., larger $p_{1}$) and sparser layer-2 networks (i.e., smaller $p_{2}$). The ${\left(b/c\right)}^{*}$ value is reasonably responsive to the changes in r (with median $\left|\text{d}{\left(b/c\right)}^{*}/\text{d}r\right|>0.4$) when $p_{1}=0.5$ and modestly so (i.e., median $\left|\text{d}{\left(b/c\right)}^{*}/\text{d}r\right|>0.16$) when $p_{1}=0.4$.

Given that most empirical contact networks are heterogeneous in terms of the node’s degree, we ran the same analysis for two-layer Barabási-Albert (BA) networks, where each new node added to the network layer L has ${\bar{m}}_{L}$ nodes, making the average degree of each network layer approximately equal to $2{\bar{m}}_{L}$ (see Methods for the procedure for generating networks). Figure 5(b) shows the proportion of network layer 1 that facilitates cooperation as the one-layer network (upper part) and the median and percentiles of $\left|\text{d}{\left(b/c\right)}^{*}/\text{d}r\right|$ (lower part). The results are qualitatively similar to those for the ER random graph. Specifically, one-layer BA networks favor cooperation in most cases if ${\bar{m}}_{1}\leq 4$. Note that ${\bar{m}}_{1}\leq 4$ does not imply particularly sparse networks because we are using networks with N=15 nodes. Furthermore, $\left|\text{d}{\left(b/c\right)}^{*}/\text{d}r\right|$ is overall larger for the two-layer BA than ER networks. In particular, at ${\bar{m}}_{1}=4$, a majority of BA networks support cooperation, and cooperation is substantially eased (i.e., median $\left|\text{d}{\left(b/c\right)}^{*}/\text{d}r\right|>0.44$) by coupling with another BA network layer subject to the constant-selection dynamics.

These results reinforce our main finding that coupling with the constant-selection dynamics can facilitate cooperation on networks.

### 3.5 Empirical networks

**Figure 6:**
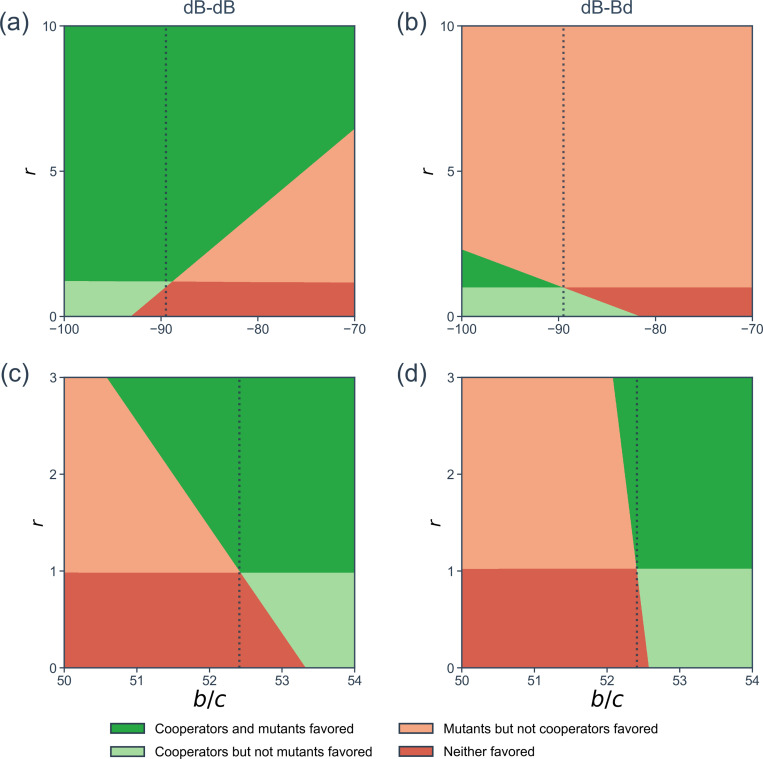
Parameter regions in which the cooperator or mutant is selected in empirical two-layer networks. The vertical lines represent the ${\left(b/c\right)}^{*}$ value for the uncoupled layer-1 network. (a) VG7, dB-dB. (b) VG7, dB-Bd. (c) LLF, dB-dB. (d) LLF, dB-Bd.

We examined two empirical two-layer networks, one with N=29 and another with N=71 (see Methods for the network description). We show in the left panels of Fig. 6 the (b/c,r) regions in which the cooperator or mutant is favored or disfavored. We obtain ${\left(b/c\right)}^{*}=-89.5$ for the VG7 two-layer network such that spite can evolve (see Fig. 6(a)). By coupling with the constant-selection dynamics layer, every change in the r value by 1 changes ${\left(b/c\right)}^{*}$ value by 3.64 (4.07%). In the case of the LLF network, cooperation can be favored, and one obtains ${\left(\frac{b}{c}\right)}^{*}=52.4$ for the uncoupled layer-1 network. When the two layers are coupled, cooperation occurs more easily if r>1 and less easily if r<1, as shown in Fig. 6(c).

### 3.6 Birth-death (Bd) rule in the constant-selection layer

Constant selection on networks has most popularly been investigated under the the Birth-death (Bd) updating rule, partly because the Bd rule amplifies the effect of selection in a majority of networks [44, 46, 47]. Therefore, we also derived the condition for favoring cooperators or mutants when layer 1 uses the dB rule and layer 2 uses the Bd rule, i.e., under the dB-Bd rule (see section S3).

Under the dB-Bd rule, because the evolutionary dynamics of the donation game is still governed by the dB rule, the condition under which cooperation is favored in layer 1 is given by Eq. (3). However, the value of $\phi_{2,0}^{\xi [1,2]}$ changes from the case of the dB-dB rule, affecting the value of ${\left(b/c\right)}^{*}$. Note that $\theta_{1}^{\xi [1]},\theta_{2}^{\xi [1]}$, and $\theta_{3}^{\xi [1]}$ remain the same because they only depend on the network structure and updating rule in layer 1.

The results for the dB-Bd rule on two-layer networks with N=6 individuals (right panel of Table 1), four sample networks (right panels of Fig. 4), and two-layer ER and BA networks (Fig. 5(c) and (d)) are qualitatively the same to those for the dB-dB rule (left panel of Table 1, middle panels of Fig. 4, and Fig. 5(a) and (b)). Notable quantitative differences are: (i) ${\left(b/c\right)}^{*}$ under the dB-Bd rule is 10.1 times more sensitive to variation in r than under the dB-dB rule in the two-layer network shown in Fig. 4(b). (ii) $\left|\text{d}{\left(b/c\right)}^{*}/\text{d}r\right|$ is substantially larger for the two-layer ER and BA networks under the dB-Bd than dB-dB rule across the network density parameters (i.e., $p_{1},p_{2},{\bar{m}}_{1}$, and ${\bar{m}}_{2}$). In the VC7 network, while ${\left(b/c\right)}^{*}$ increases as r increases under the dB-dB rule, ${\left(b/c\right)}^{*}$ decreases as r increases under the dB-Bd rule. Another observation on empirical two-layer networks is that ${\left(b/c\right)}^{*}$ is more responsive to the change in r under the dB-Bd than dB-dB rule for the VC7 network and vice versa for the LLF network. We conclude that the effect of layer-to-layer coupling on evolution of cooperation is overall similar between the dB-Bd and dB-dB rules, broadening the generality of our results.

### 3.7 “Fixed benefits, fixed costs” goods scheme

We have considered the payoff scheme in which the cooperator pays the cost c any neighbor of the cooperator receives a benefit b. This payoff scheme is the “pf goods” scheme in [38], except the difference regarding whether or not one divides the total payoff for each individual by the degree of the node, k, for normalization. To further explore generality of our results, here we consider the “ff goods” scheme [38], with which a producer (i.e., cooperator) pays a fixed cost c irrespectively of k, and each neighbor receives benefit b/k.

We derived the condition for favoring cooperators and mutants under the ff goods scheme and then examined the derived condition for the model and empirical networks that we have used (see section S9). The results are qualitatively the same as those under the pf goods scheme shown in the previous sections.

### 3.8 Fixation time

On one-layer networks, the cooperator’s fixation probability and fixation time are often in a trade-off relationship [48, 49]. The advantages of two-layer networks over one-layer networks in promoting cooperation would be compromised if fixation times are excessively longer for two-layer than one-layer networks. Therefore, we have numerically investigated fixation time in two-layer networks in some representative scenarios. To make a fair comparison between the one-layer and two-layer cases, we declared the fixation of cooperation once it is attained in layer 1 even if the resident or mutant type has not fixated in layer 2. We computed the fixation time for cooperators as the average over the runs for which the cooperator has fixated.

We show the mean fixation time for cooperators for different networks and values of b/c,r, and δ (i.e., selection strength) in section S10. For four model networks, we find that the fixation time does not substantially increase (i.e., the increase is at most ≈ 10%) in two-layer networks relative to one-layer networks including the case in which cooperation is facilitated by the two-layer networks. For the VC7 network, the fixation of cooperation needs at most 2.8 times more time when the two network layers are coupled than uncoupled.

## 4 Discussion

The donation game and constant selection are two major models whose evolutionary dynamics have been extensively studied. Both models allow substantial theoretical analysis, in particular using fixation theory, providing general and quantitative understanding. These two models have mostly been considered separately in the literature. They represent two disparate types of social interaction that humans and institutions may routinely—and simultaneously—encounter; in particular, constant-selection dynamics is equivalent to a biased voter model, a standard model of competing opinions that has been studied for many years [34–37]. We modeled this situation using a two-layer evolutionary dynamics model in which each individual is involved in the donation game in one network layer and constant-selection dynamics in the other layer. By adapting the theory developed in a recent paper that assumes different donation games in different network layers [28], we showed that coupling with the network layer undergoing constant-selection dynamics can enhance prosociality in the donation game layer, much as coupling between two donation-game layers can enhance prosociality relative to the one-layer case [28]. In other words, coupling two social dilemma games is not indispensable for enhancing prosociality; coupling with a different social dynamics can generate a spillover effect across layers that shifts the effective condition for cooperation in the dilemma layer. In our model, the condition for favoring cooperation (i.e., ${\left(b/c\right)}^{*}$) is reasonably responsive to the bias in the strength of the two opinions competing in the other layer, and conversely the donation game parameters (i.e., b and c) similarly spill over to affect the condition for favoring the mutant type. We verified the generality of our results across different networks, updating rules, and goods schemes. Extension of the present work to the case of more than two layers [23], different types of non-dilemma or social dilemma dynamics in layer 2 [26, 27, 29–31], layers of hypergraphs modeling group interactions [10, 22, 25, 50, 51], and different initial conditions may be fruitful.

Possibility of engineering the structure of one network layer to enhance cooperation in the other network layer was briefly examined in [28], where the individuals played different donation games in the different network layers. This intervention method is likely to be similarly effective at enhancing cooperation in our model, whereas clarifying how one should manipulate layer 2, in which the constant-selection dynamics takes place, awaits investigation. In addition, the correspondence between the constant-selection dynamics and the biased voter model opens further avenues for engineering cooperation. The present result implies that deploying a new opinion or fad in the unanimity of a resident opinion may enhance cooperation in the opposite network layer. One may be able to engineer the choice of individuals to which the new opinion is injected to facilitate cooperation. Furthermore, cooperation may be better enhanced by deployment of zealots, or a small number of individuals who stick to their opinion ignoring influence of other individuals [52–54]. While introduction of zealot cooperators [55] always yields fixation of cooperation in finite populations [56], introducing a zealot in opinion dynamics with the goal of enhancing cooperation may be less costly in practice than introducing a zealot cooperator.

We assumed that all the individuals generate payoffs in both layers and that the payoff summed over the two layers, $F_{i}$, is used as fecundity of the ith individual in both layers. This is a simplest case of the so-called multiplex networks, a type of multilayer networks. In fact, multilayer network research has advocated various ways in which different network layers depend on each other, driven by real-world examples [19–22]. Extending the present framework as well as prior work on donation games played in both layers [28] and constant-selection dynamics played in both layers [57] to various types of multilayer network setting with analytical rigor warrants future work. First, inter-layer coupling may be weak or unidirectional. A weaker inter-layer coupling implies that the fecundity of an individual in one layer only more weakly depends on the payoff for the same individual in the other layer [24]. The unidirectional inter-layer coupling implies that the payoff in one layer influences the fecundity in the other layer but not vice versa. Second, some nodes may be missing in one layer. Third, with a “network of network”, one assumes more than two layers and dictates which specific layer pairs interact. A network of network may be a reasonable model of human behavior when individuals playing a social dilemma game in a certain network layer may use information from some other types of social interaction corresponding to specific different network layers.

The present work has the following major limitations. First, as in previous studies relying on mathematical frameworks of fixation in multilayer networks [28, 57], it is computationally demanding to check conditions for favoring cooperators and mutants. Therefore, our present analysis is limited to small two-layer networks. However, we analyzed networks of different sizes to the best of our effort and also provided various robustness tests in dimensions other than the network size. Second, as for a plethora of work in mathematically founded studies of evolutionary game and graph theory, we assumed weak selection. Focusing on simple network structure such as the coupled complete graph and the coupled ring network to analyze the case of strong selection may be a useful analytical approach. Owing to the weak selection assumption, we refrained from over-interpreting our results throughout this manuscript. The sole over-arching message derived from our analysis is that coupling with the constant-selection dynamics, or the biased voter model dynamics, can enhance prosociality in social dilemma games played in the opposite network layer, similar to the coupling of two network layers in both of which social dilemma games are played. An important direction for future research is to test this prediction by controlled experiments and data-driven multilayer network modeling.

## Methods

### Generation of two-layer BA networks

For each layer L∈{1,2}, the initial condition of the network growth process was the complete graph on ${\bar{m}}_{L}$ nodes. Then, we grew the network by sequentially adding nodes with ${\bar{m}}_{L}$ edges according to the preferential attachment rule [58]. For each ${\bar{m}}_{L}\in \{1,2,3,4,5\}$, we generated 5 instances of BA networks with N=15 nodes for each layer, yielding 25 two-layer BA networks. We then uniformly randomly shuffled the node label of layer 2 to avoid the tendency that individuals with small indices (i.e., joining the network early in the growth process) are hubs in both layers such that the node’s degree is positively correlated between the two layers.

### Empirical two-layer networks

We have used two empirical two-layer networks. The first is the Vickers–Chan 7th Graders (VC7) network, which comprises two layers representing academic and friendship connections among 29 seventh-grade students from a school in Victoria, Australia [59]. We arbitrarily assigned the academic connection network to layer 1. The VC7 network has 126 and 152 edges in layer 1 and 2, respectively. The second network is the Lazega Law Firm (LLF) network, in which the two network layers represent professional and cooperative relationships among 71 partners within the firm [60]. We arbitrarily assigned the professional relation network to layer 1. The LLF network has 717 and 726 edges in layer 1 and 2, respectively.

## Supplementary Material

Supplement 1

## Figures and Tables

**Table 1:** Classification of all unique pairs of the two-layer network with six individuals and the initial condition. (a) dB-dB. (b) dB-Bd. The marginal distribution of $\theta_{1}-\theta_{3}$ is the same between the dB-dB and dB-Bd updating rules. This is because $\theta_{1}-\theta_{3}$ only depends on the network structure and the updating rule used in layer 1, which are common between the dB-dB and dB-Bd rules.

dB-dB	$\phi_{2,0}$			
		
$\theta_{1}-\theta_{3}$	< 0	= 0	> 0	Total

> 0	6.40%	0.00%	10.98%	17.38%
= 0	0.54%	0.00%	1.08%	1.61%
< 0	21.46%	0.00%	59.54%	81.01%

Total	28.40%	0.00%	71.60%	100.00%

> 0	8.23%	0.00%	9.15%	17.38%
= 0	0.65%	0.00%	0.96%	1.61%
< 0	38.29%	0.00%	42.72%	81.01%

Total	47.17%	0.00%	52.83%	100.00%
