# Supplementary material for: Non-dilemmatic social dynamics promote cooperation in multilayer networks
Source: ArXiv. 2026 Jan 1:arXiv:2601.00460v1. Preprint. [Version 1] (PMC12772680)
Supplement: Supplement 1 [file NIHPP2601.00460v1-supplement-1.pdf]

Supplementary Information for:

Non-dilemmatic social dynamics promote cooperation in multilayer networks

Jnanajyoti Bhaumik, Naoki Masuda

S1 Derivation of the condition under which the cooperator or mutant is favored

S1.A Replacement events and the fixation axiom

We use the notations and assumptions, particularly regarding the replacement events and fixation axiom, in Ref. [1], which was originally introduced in Ref. [2] and also used in Ref. [3]. We consider a population with two layers. In each time step, we choose a replacement event, (R, α), which consists of a pair $\left(R^{[L]}, \alpha^{[L]}\right)$ for each layer, $L \in \{1, 2\}$, where $R^{[L]}$ is the set of 'offspring' nodes that are chosen for replacement, and $\alpha^{[L]} : R^{[L]} \to \{1, \ldots, N\}$ is the offspring-to-parent map in layer L. We refer to cooperators and mutants as type A, and defectors and residents as type B. We denote by $p_{(R,\alpha)}(\boldsymbol{x})$ the probability of choosing (R, α) in state $\boldsymbol{x} \in \{0, 1\}^N \times \{0, 1\}^N$, where each entry of $\boldsymbol{x}$, denoted by $x_i^{[L]}$, is 1 and 0 if individual i has type A and B, respectively, in layer L. In each layer L, the map $\alpha^{[L]} : R^{[L]} \to \{1, \ldots, N\}$ extends to a map $\tilde{\alpha}^{[L]} : \{1, \ldots, N\} \to \{1, \ldots, N\}$ defined by $\tilde{\alpha}^{[L]}(i) = \alpha^{[L]}(i)$ if $i \in R^{[L]}$ and $\tilde{\alpha}^{[L]}(i) = i$ if $i \notin R^{[L]}$. For any state $\boldsymbol{x} \in \{0, 1\}^N \times \{0, 1\}^N$, we write $\boldsymbol{x}^{[L]} \in \{0, 1\}^N$ for the state of the population in layer $L \in \{1, 2\}$. This extension of α, denoted $\tilde{\alpha}$, gives an updated state $\boldsymbol{x}_{\tilde{\alpha}} \in \{0, 1\}^N \times \{0, 1\}^N$ defined by $(\boldsymbol{x}_{\tilde{\alpha}})_i^{[L]} = \boldsymbol{x}_{\tilde{\alpha}^{[L]}(i)}^{[L]}$. In the case of the dB and Bd processes, $R^{[L]}$ is a singleton set containing the individual i_0 to be replaced by its parent j, and we obtain $\tilde{\alpha}^{[L]}(i) = i$ if $i \neq i_0$ and $\tilde{\alpha}^{[L]}(i_0) = j$.

For $\boldsymbol{x}, \boldsymbol{y} \in \{0, 1\}^N \times \{0, 1\}^N$, we then have the state transition probability as follows:

$$P_{\boldsymbol{x} \to \boldsymbol{y}} = \sum_{(R,\alpha) \text{ s.t. } \boldsymbol{x}_{\tilde{\alpha}} = \boldsymbol{y}} p_{(R,\alpha)}(\boldsymbol{x}). \tag{S1}$$

In addition to being smooth, we assume that the replacement rule satisfies the following fixation axiom [1]:

Axiom 1 (Fixation axiom) *There exists* $(i_1, i_2) \in \{1, \ldots, N\} \times \{1, \ldots, N\}$*, an integer* $m \geq 1$*, and a sequence of replacement events* $\{(R_k, \alpha_k)\}_{k=1}^m$ *such that:*

(i) $p_{(R_k,\alpha_k)}(\boldsymbol{x}) > 0$ *for every* $k \in \{1, \ldots, m\}$ *and* $\boldsymbol{x} \in \{0, 1\}^N \times \{0, 1\}^N$.

(ii) For each $L \in \{1, 2\}$*, there exists* k_L *such that* $i_L \in R_{k_L}^{[L]}$.

(iii) For $L \in \{1, 2\}$*, we have* $\tilde{\alpha}_1^{[L]} \circ \tilde{\alpha}_2^{[L]} \circ \cdots \circ \tilde{\alpha}_m^{[L]}(j) = i_L$ *for every* $j \in \{1, \ldots, N\}$.

S1.B Weak selection and a mutation-modified Markov chain

We assume weak selection. In Ref. [1], they considered a mutation-modified Markov chain obtained by sending each absorbing state to a fixed, transient state $\boldsymbol{\xi} \in \{0, 1\}^N \times \{0, 1\}^N$ with mutation probability $u > 0$ in each time step. With probability $1 - u$, the chain remains in the same absorbing state in each time step. The benefit of this approach is that the mutation-modified Markov chain has a unique stationary

distribution that facilitates further calculations. This chain has transition probabilities given by

$$P_{\boldsymbol{x}\to\boldsymbol{y}}^{\circlearrowleft\{\boldsymbol{\xi}\}} = \begin{cases} u & \text{if } \boldsymbol{x} \in \{\boldsymbol{AA}, \boldsymbol{AB}, \boldsymbol{BA}, \boldsymbol{BB}\}, \boldsymbol{y} = \boldsymbol{\xi}, \\ (1-u)P_{\boldsymbol{x}\to\boldsymbol{y}} & \text{if } \boldsymbol{x} \in \{\boldsymbol{AA}, \boldsymbol{AB}, \boldsymbol{BA}, \boldsymbol{BB}\}, \boldsymbol{y} \neq \boldsymbol{\xi}, \\ P_{\boldsymbol{x}\to\boldsymbol{y}} & \text{if } \boldsymbol{x} \notin \{\boldsymbol{AA}, \boldsymbol{AB}, \boldsymbol{BA}, \boldsymbol{BB}\}. \end{cases} \tag{S2}$$

In Eq. (S2), $\boldsymbol{AA}$ is the state in which all the individuals are of type A in both layers, and $\boldsymbol{AB}$ is the state in which all individuals are of type A in layer 1 and type B in layer 2. States $\boldsymbol{BA}$ and $\boldsymbol{BB}$ are similarly defined. The probability that i transmits its offspring to j in layer L and in state $\boldsymbol{x}$ is

$$e_{ij}^{[L]}(\boldsymbol{x}) := \sum_{(R,\alpha) \text{ s.t. } \alpha^{[L]}(j)=i} p_{(R,\alpha)}(\boldsymbol{x}). \tag{S3}$$

We recall that δ represents the strength of selection. If $\delta = 0$, then we have neutral drift, which is denoted by $\circ$. We denote by $e_{ij}^{\circ[L]}$ the probability that i transmits its offspring to j under neutral drift in layer L. We also denote by $\pi_i^{[L]}$ the reproductive value (RV) of individual i [4–8] in layer L under neutral drift. We obtain

$$\sum_{j=1}^{N} e_{ij}^{\circ[L]} \pi_j^{[L]} = \pi_i^{[L]} \sum_{j=1}^{N} e_{ji}^{\circ[L]} \tag{S4}$$

and

$$\sum_{i=1}^{N} \pi_i^{[L]} = 1. \tag{S5}$$

The RV-weighted frequency of state $\boldsymbol{x}$ in layer L is denoted by

$$\widehat{x}^{[L]} := \sum_{i=1}^{N} \pi_i^{[L]} x_i^{[L]}. \tag{S6}$$

The change in the RV-weighted frequency in layer L due to selection in one step of the evolutionary dynamics is

$$\widehat{\Delta}_{\text{sel}}^{[L]}(\boldsymbol{x}) = \sum_{i=1}^{N} \sum_{j=1}^{N} \pi_i^{[L]} (x_j - x_i) e_{ji}^{[L]}(\boldsymbol{x}). \tag{S7}$$

The change in the RV-weighted frequency in one time step due to both mutation and selection is given by

$$\widehat{\Delta}^{[L]}(\boldsymbol{x}) = \begin{cases} u\left(\widehat{\xi}^{[L]} - 1\right) & \text{if } \boldsymbol{x}^{[L]} = \boldsymbol{A},\ \boldsymbol{x}^{[-L]} \in \{\boldsymbol{A}, \boldsymbol{B}\}, \\ u\widehat{\xi}^{[L]} & \text{if } \boldsymbol{x}^{[L]} = \boldsymbol{B},\ \boldsymbol{x}^{[-L]} \in \{\boldsymbol{A}, \boldsymbol{B}\}, \\ \widehat{\Delta}_{\text{sel}}^{[L]}(\boldsymbol{x}) & \text{if } \boldsymbol{x} \notin \{\boldsymbol{AA}, \boldsymbol{AB}, \boldsymbol{BA}, \boldsymbol{BB}\}, \end{cases} \tag{S8}$$

where $-L$ means the other layer (i.e., layer 2 if $L = 1$ and layer 1 if $L = 2$); $\widehat{\xi}^{[L]} = \sum_{i=1}^{N} \pi_i^{[L]} \xi_i^{[L]}$ is the RV-weighted frequency of type A in state $\boldsymbol{\xi}$ in layer L.

Next, we compute the expectation of the change in the RV-weighted frequency, which will enable us to compute the fixation probability of the mutation-modified Markov chain under the rare mutation condition. Because we are computing the expectation with respect to the stationary probability distribution, we obtain

$$\mathbb{E}_{\circlearrowleft(\boldsymbol{\xi})}[\widehat{\Delta}^{[L]}] = 0. \tag{S9}$$

Equation (S9) yields

$$\mathbb{E}_{\circlearrowleft(\boldsymbol{\xi})}\left[\widehat{\Delta}_{\text{sel}}^{[L]}\right] = \begin{cases} u\pi_{\circlearrowleft(\boldsymbol{\xi})}(\{\boldsymbol{A}\} \times \{\boldsymbol{A}, \boldsymbol{B}\})\left(1 - \widehat{\xi}^{[L]}\right) - u\pi_{\circlearrowleft(\boldsymbol{\xi})}(\{\boldsymbol{B}\} \times \{\boldsymbol{A}, \boldsymbol{B}\})\,\widehat{\xi}^{[L]} & \text{if } L = 1, \\ u\pi_{\circlearrowleft(\boldsymbol{\xi})}(\{\boldsymbol{A}, \boldsymbol{B}\} \times \{\boldsymbol{A}\})\left(1 - \widehat{\xi}^{[L]}\right) - u\pi_{\circlearrowleft(\boldsymbol{\xi})}(\{\boldsymbol{A}, \boldsymbol{B}\} \times \{\boldsymbol{B}\})\,\widehat{\xi}^{[L]} & \text{if } L = 2. \end{cases} \tag{S10}$$

We define $\rho_{\boldsymbol{A}}^{[L]}(\boldsymbol{\xi})$ as the fixation probability of the mutation-modified Markov chain reaching a state in which all individuals have type A in layer L when the initial state of the network is $\boldsymbol{\xi}$. By the known rare-mutation results [2, 3], we have

$$\lim_{u\to 0} \pi_{\circlearrowleft(\boldsymbol{\xi})}\left(\{\boldsymbol{A}\}\times\{\boldsymbol{A},\boldsymbol{B}\}\right) = \rho_{\boldsymbol{A}}^{[1]}(\boldsymbol{\xi}), \tag{S11}$$

$$\lim_{u\to 0} \pi_{\circlearrowleft(\boldsymbol{\xi})}\left(\{\boldsymbol{B}\}\times\{\boldsymbol{A},\boldsymbol{B}\}\right) = 1-\rho_{\boldsymbol{A}}^{[1]}(\boldsymbol{\xi}), \tag{S12}$$

$$\lim_{u\to 0} \pi_{\circlearrowleft(\boldsymbol{\xi})}\left(\{\boldsymbol{A},\boldsymbol{B}\}\times\{\boldsymbol{A}\}\right) = \rho_{\boldsymbol{A}}^{[2]}(\boldsymbol{\xi}), \tag{S13}$$

$$\lim_{u\to 0} \pi_{\circlearrowleft(\boldsymbol{\xi})}\left(\{\boldsymbol{A},\boldsymbol{B}\}\times\{\boldsymbol{B}\}\right) = 1-\rho_{\boldsymbol{A}}^{[2]}(\boldsymbol{\xi}). \tag{S14}$$

Then, we can write down the fixation probability [1] as

$$\rho_{\boldsymbol{A}}^{[L]}(\boldsymbol{\xi}) = \widehat{\xi}^{[L]} + \left.\frac{\mathrm{d}}{\mathrm{d}u}\right|_{u=0} \mathbb{E}_{\circlearrowleft(\boldsymbol{\xi})}\left[\widehat{\Delta}_{\mathrm{sel}}^{[L]}\right]. \tag{S15}$$

Because $\pi_{\circlearrowleft(\boldsymbol{\xi})}, \widehat{\xi}^{[L]}$, and u are smooth in terms of δ and u, we have [1, 2]

$$\left.\frac{\mathrm{d}}{\mathrm{d}\delta}\rho_{\boldsymbol{A}}^{[L]}(\boldsymbol{\xi})\right|_{\delta=0} = \left.\frac{\mathrm{d}}{\mathrm{d}u}\right|_{u=0} \mathbb{E}^{\circ}_{\circlearrowleft(\boldsymbol{\xi})}\left[\left.\frac{\mathrm{d}}{\mathrm{d}\delta}\right|_{\delta=0}\widehat{\Delta}_{\mathrm{sel}}^{[L]}\right]. \tag{S16}$$

Finally, for $\boldsymbol{x} \notin \{\boldsymbol{AA},\boldsymbol{AB},\boldsymbol{BA},\boldsymbol{BB}\}$, consider the rare-mutation conditional (RMC) distribution [1] given by

$$\pi_{\mathrm{RMC}(\boldsymbol{\xi})}(\boldsymbol{x}) := K \left.\frac{\mathrm{d}}{\mathrm{d}u}\right|_{u=0} \pi_{\circlearrowleft(\boldsymbol{\xi})}(\boldsymbol{x}), \tag{S17}$$

where

$$K := \left(\sum_{\boldsymbol{y}\notin\{\boldsymbol{AA},\boldsymbol{AB},\boldsymbol{BA},\boldsymbol{BB}\}} \left.\frac{\mathrm{d}}{\mathrm{d}u}\right|_{u=0} \pi_{\circlearrowleft(\boldsymbol{\xi})}(\boldsymbol{y})\right)^{-1}. \tag{S18}$$

The following lemma holds true:

Lemma 1 *For any function $\varphi : \{0,1\}^N \times \{0,1\}^N \to \mathbb{R}$,*

$$\begin{aligned}\mathbb{E}^{\circ}_{RMC(\boldsymbol{\xi})}[\varphi] = K^{\circ}\Big[&\varphi(\boldsymbol{\xi}) - \widehat{\xi}^{[1]}\widehat{\xi}^{[2]}\varphi(\boldsymbol{A},\boldsymbol{A}) - \widehat{\xi}^{[1]}(1-\widehat{\xi}^{[2]})\varphi(\boldsymbol{A},\boldsymbol{B})\\ &-(1-\widehat{\xi}^{[1]})\widehat{\xi}^{[2]}\varphi(\boldsymbol{B},\boldsymbol{A}) - (1-\widehat{\xi}^{[1]})(1-\widehat{\xi}^{[2]})\varphi(\boldsymbol{B},\boldsymbol{B})\Big]\\ &+\sum_{(R,\alpha)} p^{\circ}_{(R,\alpha)}\mathbb{E}^{\circ}_{RMC(\boldsymbol{\xi})}[\varphi_{\tilde{\alpha}}].\end{aligned} \tag{S19}$$

We recall that $\varphi_{\tilde{\alpha}}$ is the extension of the off-spring to parent map that maps the nodes in R to their parents and the nodes not in R to themselves. As indicated in Ref. [1], the proof of Eq. (S19) is an adaptation of the proof of Lemma 1 in Ref. [3]. Note that $\varphi(\boldsymbol{x})$ in Lemma 1 in Ref. [3] is replaced with function $\varphi(\boldsymbol{\xi}) - \widehat{\xi}^{[1]}\widehat{\xi}^{[2]}\varphi(\boldsymbol{A},\boldsymbol{A}) - \widehat{\xi}^{[1]}(1-\widehat{\xi}^{[2]})\varphi(\boldsymbol{A},\boldsymbol{B}) - (1-\widehat{\xi}^{[1]})\widehat{\xi}^{[2]}\varphi(\boldsymbol{B},\boldsymbol{A}) - (1-\widehat{\xi}^{[1]})(1-\widehat{\xi}^{[2]})\varphi(\boldsymbol{B},\boldsymbol{B})$ in Eq. (S19), as mentioned in Ref. [1]. For any of the four absorbing states, $\varphi(\boldsymbol{\xi}) - \widehat{\xi}^{[1]}\widehat{\xi}^{[2]}\varphi(\boldsymbol{A},\boldsymbol{A}) - \widehat{\xi}^{[1]}(1-\widehat{\xi}^{[2]})\varphi(\boldsymbol{A},\boldsymbol{B}) - (1-\widehat{\xi}^{[1]})\widehat{\xi}^{[2]}\varphi(\boldsymbol{B},\boldsymbol{A}) - (1-\widehat{\xi}^{[1]})(1-\widehat{\xi}^{[2]})\varphi(\boldsymbol{B},\boldsymbol{B})$ is equal to 0, which is the requirement for using Lemma 1 in Ref. [3]. We also use the fact that $\mathbb{E}[1] = 1$ to derive the last term on the right-hand side of Eq. (S19).

S1.C Condition for the cooperator and mutant to be selected in a general form

In this section, we derive recurrence relations for deducing conditions for selecting cooperation in the game layer (i.e., layer 1) and mutant in the constant-selection layer (i.e., layer 2). The payoff matrix of the donation game, which is used in layer 1, is given by

$$\begin{array}{c} \\ \mathrm{C} \\ \mathrm{D} \end{array} \begin{array}{c} \quad\mathrm{C} \qquad \mathrm{D} \\ \begin{pmatrix} b-c & -c \\ b & 0 \end{pmatrix} \end{array}, \tag{S20}$$

where the entries of the matrix represent the payoff to the row player. Recall that $p_{ij} = \frac{w_{ij}}{s_i}$, where w_{ij} is the (i,j) entry of the adjacency matrix of the undirected network layer 1, and s_i is the degree of the ith node in layer 1. The payoff for the kth replica node in layer 1 is given by

$$u_k^{[1]}(\boldsymbol{x}) = \sum_{\ell=1}^{N} \left(-c \cdot p_{k\ell} \cdot x_k^{[1]} + b \cdot p_{k\ell} \cdot x_\ell^{[1]} \right) = \sum_{\ell=1}^{N} \left(-C_{k\ell} \cdot x_k^{[1]} + B_{\ell k} \cdot x_\ell^{[1]} \right), \tag{S21}$$

where $C_{kl} = c \cdot p_{kl}$ and $B_{lk} = b \cdot p_{k\ell}$. We write the payoff matrix in the case of constant selection as

$$\begin{array}{c} \\ \mathrm{M} \\ \mathrm{R} \end{array} \begin{array}{c} \mathrm{M} \quad \mathrm{R} \\ \begin{pmatrix} r & r \\ 1 & 1 \end{pmatrix} \end{array}, \tag{S22}$$

where M and R stand for mutant and resident types, respectively. The payoff for the kth replica node in layer 2 is given by

$$u_k^{[2]}(\boldsymbol{x}) = x_k^{[2]} (r-1) + 1. \tag{S23}$$

The total payoff for the kth individual is given by

$$u_k(\boldsymbol{x}) = u_k^{[1]}(\boldsymbol{x}) + u_k^{[2]}(\boldsymbol{x}) = \sum_{\ell=1}^{N} \left(-C_{k\ell} \cdot x_k^{[1]} + B_{\ell k} \cdot x_\ell^{[1]} \right) + x_k^{[2]} (r-1) + 1. \tag{S24}$$

We define $\mathbf{F} = (F_1, \ldots, F_N)$, where we remind that F_i is the fecundity of the ith node. The marginal effect of the individual k's fitness on the probability that i replaces j in layer L [9] is denoted by

$$m_{k;ij}^{[L]} = \left. \frac{\partial e_{ij}^{[L]}}{\partial F_k} \right|_{\mathbf{F}=\mathbf{1}}, \tag{S25}$$

where $\mathbf{1} = (1, \ldots, 1)$. We find that

$$\left. \frac{\mathrm{d}}{\mathrm{d}\delta} e_{ij}^{[L]}(\boldsymbol{x}) \right|_{\delta=0} = \sum_{k=1}^{N} \left. \frac{\partial e_{ij}^{[L]}}{\partial F_k} \right|_{\mathbf{F}=\mathbf{1}} \cdot \left. \frac{\mathrm{d}F_k}{\mathrm{d}\delta} \right|_{\delta=0} = \sum_{k=1}^{N} m_{k;ij}^{[L]} u_k(\boldsymbol{x}) = \sum_{k=1}^{N} m_{k;ij}^{[L]} \left[u_k^{[1]}(\boldsymbol{x}) + u_k^{[2]}(\boldsymbol{x}) \right]. \tag{S26}$$

Thus, it follows from the definition of $\widehat{\Delta}_{\mathrm{sel}}^{[L]}(\boldsymbol{x})$ (i.e., Eq. (S7)) that

$$\begin{aligned} \left. \frac{\mathrm{d}}{\mathrm{d}\delta} \widehat{\Delta}_{\mathrm{sel}}^{[L]}(\boldsymbol{x}) \right|_{\delta=0} &= \sum_{i,j,k=1}^{N} \pi_i^{[L]} m_{k;ji}^{[L]} \left(x_j^{[L]} - x_i^{[L]} \right) \left[u_k^{[1]}(\boldsymbol{x}) + u_k^{[2]}(\boldsymbol{x}) \right] \\ &= \sum_{i,j,k=1}^{N} \pi_i^{[L]} m_{k;ji}^{[L]} \left(x_j^{[L]} - x_i^{[L]} \right) \left\{ \left[\sum_{\ell=1}^{N} \left(-C_{k\ell} \cdot x_k^{[1]} + B_{\ell k} \cdot x_\ell^{[1]} \right) \right] + \left[x_k^{[2]} (r-1) + 1 \right] \right\} \\ &= \sum_{i,j,k=1}^{N} \pi_i^{[L]} m_{k;ji}^{[L]} \left\{ - \left[\sum_{\ell=1}^{N} \left(x_j^{[L]} x_k^{[1]} - x_i^{[L]} x_k^{[1]} \right) C_{k\ell} - \left(x_j^{[L]} x_\ell^{[1]} - x_i^{[L]} x_\ell^{[1]} \right) B_{\ell k} \right] \right. \\ &\quad \left. + \left[\left(x_j^{[L]} x_k^{[2]} - x_i^{[L]} x_k^{[2]} \right) (r-1) + \left(x_j^{[L]} - x_i^{[L]} \right) \right] \right\}. \end{aligned} \tag{S27}$$

We denote by $x_{ij}^{\boldsymbol{\xi}[L,L']} \equiv \mathbb{E}_{\mathrm{RMC}(\boldsymbol{\xi})}\left[x_i^{[L]}x_j^{[L']}\right]$ the probability that individual i in layer L and individual j in layer L' are both of type A in the neutral RMC distribution. By taking the expectation of both sides of Eq. (S27) and combining the result with Eq. (S16) and the definition of the RMC distribution, we obtain

$$\begin{aligned}\left.\frac{\mathrm{d}}{\mathrm{d}\delta}\rho_{\boldsymbol{A}}^{[L]}(\boldsymbol{\xi})\right|_{\delta=0} =& \frac{1}{K^{\circ}}\mathbb{E}^{\circ}_{\mathrm{RMC}(\boldsymbol{\xi})}\left[\frac{\mathrm{d}}{\mathrm{d}\delta}\widehat{\Delta}_{\mathrm{sel}}^{[L]}\right]_{\delta=0} \\ =& \frac{1}{K^{\circ}}\sum_{i,j,k=1}^{N}\pi_i^{[L]}m_{k;ji}^{[L]}\left\{-\left[\sum_{\ell=1}^{N}\left(x_{jk}^{\boldsymbol{\xi}[L,1]}-x_{ik}^{\boldsymbol{\xi}[L,1]}\right)C_{k\ell}-\left(x_{j\ell}^{\boldsymbol{\xi}[L,1]}-x_{i\ell}^{\boldsymbol{\xi}[L,1]}\right)B_{\ell k}\right]\right. \\ &\left.+\left(x_{jk}^{\boldsymbol{\xi}[L,2]}-x_{ik}^{\boldsymbol{\xi}[L,2]}\right)(r-1)+\mathbb{E}_{\mathrm{RMC}(\boldsymbol{\xi})}\left[x_j^{[L]}-x_i^{[L]}\right]\right\}. \end{aligned} \tag{S28}$$

Let

$$\beta_{ij}^{\boldsymbol{\xi}[L]} := \frac{x_{ij}^{\boldsymbol{\xi}[LL]}}{K^{\circ}}, \quad L\in\{1,2\}. \tag{S29}$$

Owing to Lemma 1 in Ref. [1], $\beta_{ij}^{\boldsymbol{\xi}[1]}$ satisfies the following recurrence relation including when $i=j$:

$$\beta_{ij}^{\boldsymbol{\xi}[1]} = \xi_i^{[1]}\xi_j^{[1]}-\widehat{\xi}^{[1]}+\sum_{(R^{[1]},\alpha^{[1]})}p^{\circ}_{(R^{[1]},\alpha^{[1]})}\beta^{\boldsymbol{\xi}[1]}_{\tilde{\alpha}^{[1]}(i)\tilde{\alpha}^{[1]}(j)}. \tag{S30}$$

For the "cross terms" which are associated to the two layers jointly, we let

$$\gamma_{ij}^{\boldsymbol{\xi}[12]} := \frac{x_{ij}^{\boldsymbol{\xi}[12]}}{K^{\circ}} \tag{S31}$$

and

$$\gamma_{ij}^{\boldsymbol{\xi}[21]} := \frac{x_{ij}^{\boldsymbol{\xi}[21]}}{K^{\circ}}. \tag{S32}$$

Owing to Lemma 1 in Ref. [1], $\gamma_{ij}^{\boldsymbol{\xi}[12]}$ satisfies the recurrence relation given by

$$\gamma_{ij}^{\boldsymbol{\xi}[12]} = \xi_i^{[1]}\xi_j^{[2]}-\widehat{\xi}^{[1]}\widehat{\xi}^{[2]}+\sum_{(R^{[1]},\alpha^{[1]}),(R^{[2]},\alpha^{[2]})}p^{\circ}_{(R^{[1]},\alpha^{[1]})}p^{\circ}_{(R^{[2]},\alpha^{[2]})}\gamma^{\boldsymbol{\xi}[1,2]}_{\tilde{\alpha}^{[1]}(i)\tilde{\alpha}^{[2]}(j)} \tag{S33}$$

including when $i=j$. Equation (S33) implies that $\gamma_{ij}^{\boldsymbol{\xi}[12]}=\gamma_{ij}^{\boldsymbol{\xi}[21]}$. To compute $\mathbb{E}_{\mathrm{RMC}(\boldsymbol{\xi})}\left[x_j^{[L]}-x_i^{[L]}\right]$ on the right-hand side of Eq. (S28), we let

$$\eta_i^{\boldsymbol{\xi}[1]} := \mathbb{E}_{\mathrm{RMC}(\boldsymbol{\xi})}\left[\frac{x_i^{[1]}}{K^{\circ}}\right]. \tag{S34}$$

Owing to Lemma 1 in Ref. [1], $\eta_1^{\boldsymbol{\xi}[1]}$, …, $\eta_N^{\boldsymbol{\xi}[1]}$ satisfy the recurrence relation given by

$$\eta_i^{\boldsymbol{\xi}[1]} = \xi_i^{[1]}-\widehat{\xi}^{[1]}\widehat{\xi}^{[2]}-\widehat{\xi}^{[1]}\left(1-\widehat{\xi}^{[2]}\right)+\sum_{(R^{[1]},\alpha^{[1]})}p^{\circ}_{(R^{[1]},\alpha^{[1]})}\eta^{\boldsymbol{\xi}[1]}_{\tilde{\alpha}^{[1]}(i)}. \tag{S35}$$

By setting $L=1$ in Eq. (S28) and using Eqs. (S29), (S31), and (S34), we obtain

$$\begin{aligned}\left.\frac{\mathrm{d}}{\mathrm{d}\delta}\rho_{\boldsymbol{A}}^{[1]}(\boldsymbol{\xi})\right|_{\delta=0} = \sum_{i,j,k=1}^{N}\pi_i^{[1]}m_{k;ji}^{[1]}&\left\{-\left[\sum_{\ell=1}^{N}\left(\beta_{jk}^{\boldsymbol{\xi}[1]}-\beta_{ik}^{\boldsymbol{\xi}[1]}\right)C_{k\ell}-\left(\beta_{jl}^{\boldsymbol{\xi}[1]}-\beta_{il}^{\boldsymbol{\xi}[1]}\right)B_{\ell k}\right]\right. \\ &\left.+\left[\left(\gamma_{jk}^{\boldsymbol{\xi}[1,2]}-\gamma_{ik}^{\boldsymbol{\xi}[1,2]}\right)(r-1)+\left(\eta_j^{\boldsymbol{\xi}[1]}-\eta_i^{\boldsymbol{\xi}[1]}\right)\right]\right\}. \end{aligned} \tag{S36}$$

If $\beta_{ij}^{\boldsymbol{\xi}[1]}$ is a solution to Eq. (S30), then $\beta_{ij}^{\boldsymbol{\xi}[1]} + C'$ is also a solution to Eq. (S30) for any constant C'. Similarly, if $\gamma_{ij}^{\boldsymbol{\xi}[1,2]}$ is a solution to Eq. (S33), then $\gamma_{ij}^{\boldsymbol{\xi}[1,2]} + C''$ is also a solution to Eq. (S33) for any constant C''. The authors of Ref. [1] also pointed out that it does not matter which solution is used because only the differences of $\beta_{ij}^{\boldsymbol{\xi}[1]}$ and $\gamma_{ij}^{\boldsymbol{\xi}[1,2]}$ are used in Eq. (S36). By the fixation axiom in Refs. [1–3], the space of solutions is two-dimensional. In Ref. [1], they enforced two additional arbitrary constraints, which altogether ensure that the solution is unique. We impose these conditions, which are

$$\sum_{i=1}^{N} \pi_i^{[1]} \beta_{ii}^{\boldsymbol{\xi}[1]} = 0 \tag{S37}$$

and

$$\sum_{i=1}^{N} \pi_i^{[1]} \gamma_{ii}^{\boldsymbol{\xi}[1,2]} = 0. \tag{S38}$$

In our case, we have the term $\eta_j^{\boldsymbol{\xi}[1]} - \eta_i^{\boldsymbol{\xi}[1]}$, which is not present in Ref. [1]. Therefore, we also impose that $\sum_{i=1}^{N} \eta_i^{\boldsymbol{\xi}[1]} = 0$ to ensure uniqueness of the solution, which is again justified because only the pairwise difference $\eta_j^{\boldsymbol{\xi}[1]} - \eta_i^{\boldsymbol{\xi}[1]}$ matters.

Equation (S15) implies that selection favors cooperation in the limit of weak selection when starting from state $\boldsymbol{\xi}$ if and only if

$$\left.\frac{\mathrm{d}}{\mathrm{d}\delta} \rho_{\boldsymbol{A}}^{[1]}(\boldsymbol{\xi})\right|_{\delta=0} > 0. \tag{S39}$$

By substituting Eq. (S36) in Eq. (S39), we obtain

$$\begin{aligned} \sum_{i,j,k=1}^{N} \pi_i^{[1]} m_{k,ji}^{[1]} \Bigg\{ & - \left[\sum_{\ell=1}^{N} \left(\beta_{jk}^{\boldsymbol{\xi}[1]} - \beta_{ik}^{\boldsymbol{\xi}[1]} \right) C_{k\ell} - \left(\beta_{jl}^{\boldsymbol{\xi}[1]} - \beta_{il}^{\boldsymbol{\xi}[1]} \right) B_{\ell k} \right] \\ & + \left[\left(\gamma_{jk}^{\boldsymbol{\xi}[1,2]} - \gamma_{ik}^{\boldsymbol{\xi}[1,2]} \right) (r-1) + \left(\eta_j^{\boldsymbol{\xi}[1]} - \eta_i^{\boldsymbol{\xi}[1]} \right) \right] \Bigg\} > 0. \end{aligned} \tag{S40}$$

S2 Evolution of the cooperator and mutant under the dB-dB rule

In this section, we derive the condition under which the cooperator is favored in layer 1 and that under which the mutant is favored in layer 2, assuming the dB rule in both layers, i.e., dB-dB rule. The derivation amounts to calculating individual quantities in Eq. (S40) and simplifying the expressions to facilitate interpretation and numerical computations.

S2.A Preliminaries

For the dB rule, we obtain

$$e_{ji}^{[1]} = \frac{1}{N} \frac{w_{ij}^{[1]} F_i(x)}{\sum_{\ell=1}^{N} w_{\ell i} F_\ell(x)}. \tag{S41}$$

By substituting Eq. (S41) in Eq. (S25), we obtain

$$\begin{aligned} m_{k;ji} = \left.\frac{\partial e_{ji}}{\partial F_k}\right|_{\mathbf{F}=\mathbf{1}} &= \left.\frac{\partial}{\partial F_k} \frac{1}{N} \frac{w_{ji} F_j(x)}{\sum_{\ell=1}^{N} w_{\ell i} F_\ell(x)}\right|_{\mathbf{F}=\mathbf{1}} \\ &= \begin{cases} -\frac{1}{N} w_{ji} F_j(x) \frac{w_{ki}}{\left[\sum_{\ell=1}^{N} w_{\ell i} F_\ell(x)\right]^2}\Big|_{\mathbf{F}=\mathbf{1}} & \text{if } j \neq k \\ \frac{1}{N} w_{ki} \frac{\left[\sum_{\ell=1}^{N} w_{\ell i} F_\ell(x)\right] - F_k(x) w_{ki}}{\left[\sum_{\ell=1}^{N} w_{\ell i} F_\ell(x)\right]^2}\Big|_{\mathbf{F}=\mathbf{1}} & \text{if } j = k \end{cases} \\ &= \begin{cases} -\frac{1}{N} w_{ji} \frac{w_{ki}}{\left(\sum_{\ell=1}^{N} w_{\ell i}\right)^2} & \text{if } j \neq k \\ \frac{1}{N} w_{ki} \frac{\left(\sum_{\ell=1}^{N} w_{\ell i}\right) - w_{ki}}{\left[\sum_{\ell=1}^{N} w_{\ell i} F_\ell(x)\right]^2} & \text{if } j = k \end{cases} \\ &= \begin{cases} -\frac{1}{N} p_{ij} p_{ik} & \text{if } j \neq k \\ \frac{1}{N} p_{ij} (1 - p_{ik}) & \text{if } j = k \end{cases} \\ &= \frac{1}{N} p_{ij} \left(\delta_{j,k} - p_{ik}\right). \end{aligned} \tag{S42}$$

We have dropped the superscript $[L]$ for $m_{k;ji}^{[L]}$ and $w_{ij}^{[L]}$ in Eq. (S42) because the calculations are identical for any L.

S2.B Conditions for the cooperator to be selected

To compute the condition under which the cooperator is favored, Eq. (S40), we introduce a short-hand notation

$$f_{ijk} \equiv -\beta_{ij}^{\boldsymbol{\xi}[1]} C_{jk} + \beta_{ik}^{\boldsymbol{\xi}[1]} B_{kj} + \gamma_{ij}^{\boldsymbol{\xi}[1,2]} (r-1) + \eta_i^{\boldsymbol{\xi}[1]}. \tag{S43}$$

By substituting Eq. (S43) in Eq. (S36), we obtain

$$
\begin{aligned}
\left.\frac{\mathrm{d}}{\mathrm{d}\delta}\rho_{\boldsymbol{A}}^{[1]}(\boldsymbol{\xi})\right|_{\delta=0} &= \sum_{i,j,k,\ell=1}^{N} \pi_i^{[1]} m_{k;ji}^{[1]} (f_{jkl} - f_{ikl}) \\
&= \sum_{i,j,k,\ell=1}^{N} \pi_i^{[1]} \frac{1}{N} p_{ij}^{[1]} (\delta_{j,k} - p_{ik}^{[1]})(f_{jkl} - f_{ikl}) \\
&= \frac{1}{N} \sum_{i,j,k,\ell=1}^{N} \pi_i^{[1]} p_{ij}^{[1]} \delta_{j,k} f_{jkl} - \frac{1}{N} \sum_{i,j,k,\ell=1}^{N} \pi_i^{[1]} p_{ij}^{[1]} p_{ik}^{[1]} f_{jkl} \\
&\quad - \frac{1}{N} \sum_{i,j,k,\ell=1}^{N} \pi_i^{[1]} p_{ij}^{[1]} \delta_{j,k} f_{ikl} + \frac{1}{N} \sum_{i,j,k,\ell=1}^{N} \pi_i^{[1]} p_{ij}^{[1]} p_{ik}^{[1]} f_{ikl} \\
&= \frac{1}{N} \sum_{i,j,\ell=1}^{N} \pi_i^{[1]} p_{ij}^{[1]} f_{jjl} - \frac{1}{N} \sum_{i,j,k,\ell=1}^{N} \pi_i^{[1]} p_{ji}^{[1]} p_{ik}^{[1]} f_{jkl} \\
&\quad - \frac{1}{N} \sum_{i,j,\ell=1}^{N} \pi_i^{[1]} p_{ij}^{[1]} f_{ijl} + \frac{1}{N} \sum_{i,k,\ell=1}^{N} \pi_i^{[1]} \left(\sum_{j=1}^{N} p_{ij}^{[1]} \right) p_{ik}^{[1]} f_{ikl} \\
&= \frac{1}{N} \sum_{j,\ell=1}^{N} \pi_j^{[1]} \left(\sum_{i=1}^{N} p_{ji}^{[1]} \right) f_{jjl} - \frac{1}{N} \sum_{j,k,\ell=1}^{N} \pi_j^{[1]} \left(p^{[1]}\right)_{jk}^{(2)} f_{jkl} \\
&\quad \cancel{- \frac{1}{N} \sum_{i,j,\ell=1}^{N} \pi_i^{[1]} p_{ij}^{[1]} f_{ijl}} + \cancel{\frac{1}{N} \sum_{i,k,\ell=1}^{N} \pi_i^{[1]} p_{ik}^{[1]} f_{ikl}} \\
&= \frac{1}{N} \sum_{j,\ell=1}^{N} \pi_j^{[1]} f_{jjl} - \frac{1}{N} \sum_{i,j,\ell=1}^{N} \pi_i^{[1]} \left(p^{[1]}\right)_{ij}^{(2)} f_{ijl} \\
&= \frac{1}{N} \sum_{i,\ell=1}^{N} \pi_i^{[1]} \left[-\beta_{ii}^{\boldsymbol{\xi}[1]} C_{il} + \beta_{il}^{\boldsymbol{\xi}[1]} B_{li} + \gamma_{ii}^{\boldsymbol{\xi}[1,2]}(r-1) + \eta_i^{\boldsymbol{\xi}[1]} \right] \\
&\quad - \frac{1}{N} \sum_{i,j,\ell=1}^{N} \pi_i^{[1]} \left(p^{[1]}\right)_{ij}^{(2)} \left[-\beta_{ij}^{\boldsymbol{\xi}[1]} C_{jl} + \beta_{il}^{\boldsymbol{\xi}[1]} B_{lj} + \gamma_{ij}^{\boldsymbol{\xi}[1,2]}(r-1) + \eta_i^{\boldsymbol{\xi}[1]} \right],
\end{aligned}
\tag{S44}
$$

where $\left(p^{[1]}\right)_{ij}^{(n)}$ is the probability that the random walker in layer 1 moves from replica node i to replica node j in n steps. To derive the first equality in Eq. (S44), we used Eq. (S42). To derive the third equality in Eq. (S44), we used $\pi_i^{[1]} p_{ij}^{[1]} = \pi_j^{[1]} p_{ji}^{[1]}$, $\forall\ i, j$. Equation (S44) implies that $\left.\frac{\mathrm{d}}{\mathrm{d}\delta}\rho_{\boldsymbol{A}}^{[1]}(\boldsymbol{\xi})\right|_{\delta=0} > 0$ holds true if and only if

$$
\begin{aligned}
&\sum_{i=1}^{N} \pi_i^{[1]} \sum_{\ell=1}^{N} \left[-\beta_{ii}^{\boldsymbol{\xi}[1]} C_{il} + \beta_{il}^{\boldsymbol{\xi}[1]} B_{li} + \gamma_{ii}^{\boldsymbol{\xi}[1,2]} (r-1) + \eta_i^{\boldsymbol{\xi}[1]} \right] > \\
&\sum_{i,j=1}^{N} \pi_i^{[1]} \left(p^{[1]}\right)_{ij}^{(2)} \sum_{\ell=1}^{N} \left[-\beta_{ij}^{\boldsymbol{\xi}[1]} C_{jl} + \beta_{il}^{\boldsymbol{\xi}[1]} B_{lj} + \gamma_{ij}^{\boldsymbol{\xi}[1,2]} (r-1) + \eta_i^{\boldsymbol{\xi}[1]} \right].
\end{aligned}
\tag{S45}
$$

By substituting $C_{kl} = c \cdot p_{kl}^{[1]}$ and $B_{lk} = b \cdot p_{lk}^{[1]}$ in Eq. (S45), we obtain

$$\sum_{i=1}^{N} \pi_i^{[1]} \sum_{\ell=1}^{N} \left[-\beta_{ii}^{\boldsymbol{\xi}[1]} c \cdot p_{il}^{[1]} + \beta_{il}^{\boldsymbol{\xi}[1]} b \cdot p_{il}^{[1]} + \gamma_{ii}^{\boldsymbol{\xi}[1,2]} (r-1) + \eta_i^{\boldsymbol{\xi}[1]} \right] >$$
$$\sum_{i,j=1}^{N} \pi_i^{[1]} \left(p^{[1]} \right)_{ij}^{(2)} \sum_{\ell=1}^{N} \left[-\beta_{ij}^{\boldsymbol{\xi}[1]} c \cdot p_{jl}^{[1]} + \beta_{il}^{\boldsymbol{\xi}[1]} b \cdot p_{jl}^{[1]} + \gamma_{ij}^{\boldsymbol{\xi}[1,2]} (r-1) + \eta_i^{\boldsymbol{\xi}[1]} \right]. \tag{S46}$$

To simplify Eq. (S46), we define

$$\theta_n^{\boldsymbol{\xi}[1]} := \sum_{i,j=1}^{N} \pi_i^{[1]} \left(p^{[1]} \right)_{ij}^{(n)} \beta_{ij}^{\boldsymbol{\xi}[1]} \tag{S47}$$

and

$$\phi_{n,m}^{\boldsymbol{\xi}[1,2]} := \sum_{i,j=1}^{N} \pi_i^{[1]} \left(p^{[1,2]} \right)_{ij}^{(n,m)} \gamma_{ij}^{\boldsymbol{\xi}[1,2]}, \tag{S48}$$

where $\left(p^{[1,2]} \right)_{ij}^{(n,m)}$ is the probability that the random walker moves from replica node i to replica node j when the first n steps of the random walk occur in layer 1 and the subsequent m steps occur in layer 2. By substituting Eqs. (S47) and (S48) in Eq. (S46) and using the fact that

$$\sum_{i,j=1}^{N} \pi_i^{[1]} \left(p^{[1]} \right)_{ij}^{(n)} \eta_i^{\boldsymbol{\xi}[1]} = \sum_{i=1}^{N} \pi_i^{[1]} \eta_i^{\boldsymbol{\xi}[1]} \sum_{j=1}^{N} \left(p^{[1]} \right)_{ij}^{(n)} = \sum_{i=1}^{N} \pi_i^{[1]} \eta_i^{\boldsymbol{\xi}[1]} = 0 \tag{S49}$$

for any n, we obtain

$$c\theta_2^{\boldsymbol{\xi}[1]} + b \left(\theta_1^{\boldsymbol{\xi}[1]} - \theta_3^{\boldsymbol{\xi}[1]} \right) - (r-1) \phi_{2,0}^{\boldsymbol{\xi}[1,2]} > 0, \tag{S50}$$

which is Eq. (3) in the main text. It should be noted that $\eta_i^{\boldsymbol{\xi}[1]}$ in Eq. (S46) has been canceled out.

Next, we solve the recurrence equations Eqs. (S30), (S33), and (S35) to determine $\theta_n^{\boldsymbol{\xi}[1]}$, $n \in \{1,2,3\}$ and $\phi_{n,m}^{\boldsymbol{\xi}[1,2]}$ through Eq. (S47) and (S48), respectively. The rest of the derivation is identical to section 2.1.1 of the ESM for Ref. [1] because these recurrences were derived in Ref. [1] for any dynamics using the dB rule, except Eq. (S57). Therefore, we omit some details. Note that Eq. (S57) contains the recurrence for the η term, which is absent in their case. For $i = j$, Eq. (S30) reduces to

$$\beta_{ii}^{\boldsymbol{\xi}[1]} = \xi_i^{[1]} - \widehat{\xi}^{[1]} + \frac{1}{N} \sum_{k=1}^{N} p_{ik}^{[1]} \beta_{kk}^{\boldsymbol{\xi}[1]} + \left(1 - \frac{1}{N} \right) \beta_{ii}^{\boldsymbol{\xi}[1]}, \tag{S51}$$

which gives

$$\beta_{ii}^{\boldsymbol{\xi}[1]} = N \left(\xi_i^{[1]} - \widehat{\xi}^{[1]} \right) + \sum_{k=1}^{N} p_{ik}^{[1]} \beta_{kk}^{\boldsymbol{\xi}[1]}. \tag{S52}$$

For $i \neq j$, we obtain

$$\beta_{ij}^{\boldsymbol{\xi}[1]} = \xi_i^{[1]} \xi_j^{[1]} - \widehat{\xi}^{[1]} + \frac{1}{N} \sum_{k=1}^{N} p_{ik}^{[1]} \beta_{kj}^{\boldsymbol{\xi}[1]} + \frac{1}{N} \sum_{k=1}^{N} p_{jk}^{[1]} \beta_{ik}^{\boldsymbol{\xi}[1]} + \left(1 - \frac{2}{N} \right) \beta_{ij}^{\boldsymbol{\xi}[1]}, \tag{S53}$$

which gives

$$\beta_{ij}^{\boldsymbol{\xi}[1]} = \frac{N}{2} \left(\xi_i^{[1]} \xi_j^{[1]} - \widehat{\xi}^{[1]} \right) + \frac{1}{2} \sum_{k=1}^{N} p_{ik}^{[1]} \beta_{kj}^{\boldsymbol{\xi}[1]} + \frac{1}{2} \sum_{k=1}^{N} p_{jk}^{[1]} \beta_{ik}^{\boldsymbol{\xi}[1]}. \tag{S54}$$

For $i, j \in \{1, \dots, N\}$, Eq. (S33) reduces to

$$\gamma_{ij}^{\boldsymbol{\xi}[1,2]} = \xi_i^{[1]}\xi_j^{[2]} - \widehat{\xi}^{[1]}\widehat{\xi}^{[2]} + \frac{1}{N^2}\sum_{k_1,k_2=1}^{N} p_{ik_1}^{[1]} p_{jk_2}^{[2]} \gamma_{k_1k_2}^{\boldsymbol{\xi}[1,2]} + \frac{1}{N}\left(1-\frac{1}{N}\right)\sum_{k_1=1}^{N} p_{ik_1}^{[1]}\gamma_{k_1j}^{\boldsymbol{\xi}[1,2]} + \frac{1}{N}\left(1-\frac{1}{N}\right)\sum_{k_2=1}^{N} p_{jk_2}^{[2]}\gamma_{ik_2}^{\boldsymbol{\xi}[1,2]} + \left(1-\frac{1}{N}\right)^2 \gamma_{ij}^{\boldsymbol{\xi}[1,2]}, \tag{S55}$$

which gives

$$\gamma_{ij}^{\boldsymbol{\xi}[1,2]} = \frac{N^2}{2N-1}\left(\xi_i^{[1]}\xi_j^{[2]} - \widehat{\xi}^{[1]}\widehat{\xi}^{[2]}\right) + \frac{1}{2N-1}\sum_{k_1,k_2=1}^{N} p_{ik_1}^{[1]} p_{jk_2}^{[2]} \gamma_{k_1k_2}^{\boldsymbol{\xi}[1,2]} + \frac{N-1}{2N-1}\sum_{k_1=1}^{N} p_{ik_1}^{[1]}\gamma_{k_1j}^{\boldsymbol{\xi}[1,2]} + \frac{N-1}{2N-1}\sum_{k_2=1}^{N} p_{jk_2}^{[2]}\gamma_{ik_2}^{\boldsymbol{\xi}[1,2]}. \tag{S56}$$

For $i \in \{1, \dots, N\}$, Eq. (S35) reduces to

$$\eta_i^{\boldsymbol{\xi}[1]} = \xi_i^{[1]} - \widehat{\xi}^{[1]}\widehat{\xi}^{[2]} - \widehat{\xi}^{[1]}\left(1-\widehat{\xi}^{[2]}\right) + \frac{1}{N}\sum_{k=1}^{N} p_{ik}^{[1]}\eta_k^{\boldsymbol{\xi}[1]} + \left(1-\frac{1}{N}\right)\eta_i^{\boldsymbol{\xi}[1]}, \tag{S57}$$

which gives

$$\eta_i^{\boldsymbol{\xi}[1]} = N\left[\xi_i^{[1]} - \widehat{\xi}^{[1]}\widehat{\xi}^{[2]} - \widehat{\xi}^{[1]}\left(1-\widehat{\xi}^{[2]}\right)\right] + \sum_{k=1}^{N} p_{ik}^{[1]}\eta_k^{\boldsymbol{\xi}[1]}. \tag{S58}$$

By solving the recurrences given by Eqs. (S52), (S54), (S56), and (S58), and substituting the solutions in Eqs. (S47) and (S48) and the obtained $\theta_n^{\boldsymbol{\xi}[1]}$ and $\phi_{n,m}^{\boldsymbol{\xi}[1,2]}$ in Eq. (S50), we obtain the conditions under which selection favors cooperation.

S2.C Conditions for the mutant to be selected

To avoid complication of the equations, in this section, we swap the two layers such that constant selection occurs in layer 1 and the donation game occurs in layer 2. Then, Eq. (S36) becomes

$$\left.\frac{\mathrm{d}}{\mathrm{d}\delta}\rho_{\boldsymbol{A}}^{[1]}(\boldsymbol{\xi})\right|_{\delta=0} = \sum_{i,j,k=1}^{N}\frac{1}{N}\pi_i^{[1]} m_{k;ji}^{[1]}\left\{\left[\left(\beta_{jk}^{\boldsymbol{\xi}[1]} - \beta_{ik}^{\boldsymbol{\xi}[1]}\right)(r-1) + \left(\eta_j^{\boldsymbol{\xi}[1]} - \eta_i^{\boldsymbol{\xi}[1]}\right)\right] + \sum_{\ell=1}^{N}\left[-\left(\gamma_{jk}^{\boldsymbol{\xi}[1,2]} - \gamma_{ik}^{\boldsymbol{\xi}[1,2]}\right)C_{k\ell} + \left(\gamma_{j\ell}^{\boldsymbol{\xi}[1,2]} - \gamma_{i\ell}^{\boldsymbol{\xi}[1,2]}\right)B_{\ell k}\right]\right\}. \tag{S59}$$

Similarly, Eq. (S46) becomes

$$\sum_{i=1}^{N}\pi_i^{[1]}\sum_{\ell=1}^{N}\left[\beta_{ii}^{\boldsymbol{\xi}[1]}(r-1) + \eta_i^{\boldsymbol{\xi}[1]} - \gamma_{ii}^{\boldsymbol{\xi}[1,2]} c p_{k\ell}^{[2]} + \gamma_{i\ell}^{\boldsymbol{\xi}[1,2]} b p_{i\ell}^{[2]}\right] > \sum_{i,j=1}^{N}\pi_i^{[1]}\left(p^{[1]}\right)_{ij}^{(2)}\sum_{\ell=1}^{N}\left[\beta_{ij}^{\boldsymbol{\xi}[1]}(r-1) + \eta_i^{\boldsymbol{\xi}[1]} - \gamma_{ij}^{\boldsymbol{\xi}[1,2]} c p_{j\ell}^{[2]} + \gamma_{i\ell}^{\boldsymbol{\xi}[1,2]} b p_{j\ell}^{[2]}\right]. \tag{S60}$$

By carrying out calculations similar to those in Ref. [1], we find that Equation (S60) simplifies to

$$-(r-1)\,\theta_2^{\boldsymbol{\xi}[1]} + c\phi_{2,0}^{\boldsymbol{\xi}[1,2]} + b\left(\phi_{0,1}^{\boldsymbol{\xi}[1,2]} - \phi_{2,1}^{\boldsymbol{\xi}[1,2]}\right) > 0, \tag{S61}$$

which is Eq. (4) in the main text.

S3 Evolution of cooperator and mutant under the dB-Bd rule

In this section, we derive the conditions for the cooperation and mutant type to be favored when updating occurs according to the death-Birth rule in the game layer (i.e., layer 1) and the Birth-death rule in constant-selection layer (i.e., layer 2), i.e., under the dB-Bd rule.

S3.A Preliminaries

For the Bd rule, we obtain

$$e_{ji}^{[2]} = \frac{w_{ji}}{\sum_{\ell=1}^{N} w_{j\ell}^{[2]}} \cdot \frac{F_j(x)}{\sum_{\ell=1}^{N} F_\ell(x)} \tag{S62}$$

for layer 2. By substituting Eq. (S62) in Eq. (S25), we obtain

$$\begin{aligned} m_{k;ji}^{[2]} = \left.\frac{\partial e_{ji}}{\partial F_k}\right|_{\mathbf{F}=\mathbf{1}} &= \frac{w_{ji}^{[2]}}{\sum_{\ell=1}^{N} w_{j\ell}^{[2]}} \left.\frac{\partial}{\partial F_k}\frac{F_j(x)}{\sum_{\ell=1}^{N} F_\ell(x)}\right|_{\mathbf{F}=\mathbf{1}} \\ &= \begin{cases} p_{ji}^{[2]} F_j(x) \left.\frac{-1}{\left[\sum_{\ell=1}^{N} F_\ell(x)\right]^2}\right|_{\mathbf{F}=\mathbf{1}} & \text{if } j \neq k \\ p_{ji}^{[2]} \left.\frac{\left[\sum_{\ell=1}^{N} F_\ell(x)\right] - F_k(x)}{\left[\sum_{\ell=1}^{N} w_{\ell i} F_\ell(x)\right]^2}\right|_{\mathbf{F}=\mathbf{1}} & \text{if } j = k \end{cases} \\ &= \begin{cases} -\frac{1}{N} p_{ji}^{[2]} & \text{if } j \neq k \\ \frac{N-1}{N^2} p_{ji}^{[2]} & \text{if } j = k \end{cases} \\ &= \frac{1}{N}\left(\delta_{j,k} - \frac{1}{N}\right) p_{ji}^{[2]}. \end{aligned} \tag{S63}$$

S3.B Conditions for the cooperator to be selected

Because layer 1 uses the dB rule, the condition under which the cooperator is selected remains in the same form, i.e., Eq. (S50). Furthermore, $\theta_n^{\boldsymbol{\xi}[1]}$, $n \in \{1,2,3\}$ remains the same as that under the dB-dB rule. This is because, in Eq. (S47), $\left(p^{[1]}\right)_{ij}^{(n)}$ only depends on the network structure in layer 1, $\pi_i^{[1]}$ depends on the network structure in layer 1 and the updating rule used in layer 1, and $\beta_{ij}^{\boldsymbol{\xi}[1]}$ only depends on the network structure and updating rule in layer 1. For obtaining $\beta_{ij}^{\boldsymbol{\xi}[1]}$, the recurrence equations to be used are Eqs. (S52) and (S54).

In contrast, the value of $\phi_{2,0}^{\boldsymbol{\xi}[1,2]}$ changes from the case of the dB-dB rule because $\gamma_{ij}^{\boldsymbol{\xi}[1,2]}$ in Eq. (S48) depends on the updating rule in layer 2 as well as the network structure of both layers. It should be noted that $\left(p^{[1,2]}\right)_{ij}^{(n,m)}$ still only depends on the network structure in both layers and hence is not influenced by the change in the updating rule in layer 2. We derive $\gamma_{ij}^{\boldsymbol{\xi}[1,2]}$ in section S3.C (see the self-consistent equation (S73) for $\gamma_{ij}^{\boldsymbol{\xi}[1,2]}$; note that $\gamma_{ij}^{\boldsymbol{\xi}[1,2]} = \gamma_{ij}^{\boldsymbol{\xi}[2,1]}$).

S3.C Conditions for the mutant to be selected

Similarly to Eq. (S43), we set

$$f_{ijk} \equiv -\gamma_{ij}^{\boldsymbol{\xi}[2,1]} C_{jk} + \gamma_{ik}^{\boldsymbol{\xi}[2,1]} B_{kj} + \beta_{ij}^{\boldsymbol{\xi}[2]}(r-1) + \eta_i^{\boldsymbol{\xi}[2]}. \tag{S64}$$

Note that the reproductive value under the Bd rule, which is assumed for layer 2 here, is given by [10,11,38]

$$\pi_i^{[2]} = \frac{\left(s_i^{[2]}\right)^{-1}}{\sum_{\ell=1}^{N}\left(s_\ell^{[2]}\right)^{-1}}. \tag{S65}$$

Therefore, we obtain

$$\begin{aligned}\left.\frac{\mathrm{d}}{\mathrm{d}\delta}\rho_{\boldsymbol{A}}^{[2]}(\boldsymbol{\xi})\right|_{\delta=0}=&\sum_{i,j,k,\ell=1}^{N}\pi_i^{[2]}m_{k;ji}^{[2]}\left(f_{jk\ell}-f_{ik\ell}\right)\\=&\frac{1}{N}\sum_{i,j=1}^{N}\pi_i^{[2]}p_{ji}^{[2]}\sum_{\ell=1}^{N}\left[-\gamma_{ii}^{\boldsymbol{\xi}[2,1]}C_{i\ell}+\gamma_{i\ell}^{\boldsymbol{\xi}[2,1]}B_{\ell i}+\beta_{ii}^{\boldsymbol{\xi}[2]}\left(r-1\right)+\eta_i^{\boldsymbol{\xi}[2]}\right]\\&-\frac{1}{N}\sum_{i,j=1}^{N}\pi_i^{[2]}p_{ji}^{[2]}\sum_{\ell=1}^{N}\left[-\gamma_{ik}^{\boldsymbol{\xi}[2,1]}C_{k\ell}+\gamma_{i\ell}^{\boldsymbol{\xi}[2,1]}B_{\ell j}+\beta_{ik}^{\boldsymbol{\xi}[2]}\left(r-1\right)+\eta_i^{\boldsymbol{\xi}[2]}\right].\end{aligned}\tag{S66}$$

For detailed derivation of Eq. (S66), see Eq. (SI.60) in Ref. [1]. Equation (S66) implies that $\left.\frac{\mathrm{d}}{\mathrm{d}\delta}\rho_{\boldsymbol{A}}^{[2]}(\boldsymbol{\xi})\right|_{\delta=0}>0$ if and only if

$$\begin{aligned}&\sum_{i,j=1}^{N}\pi_i^{[2]}p_{ji}^{[2]}\sum_{\ell=1}^{N}\left[-\gamma_{ii}^{\boldsymbol{\xi}[2,1]}cp_{i\ell}^{[1]}+\gamma_{i\ell}^{\boldsymbol{\xi}[2,1]}bp_{i\ell}^{[1]}+\beta_{ii}^{\boldsymbol{\xi}[2]}\left(r-1\right)+\eta_i^{\boldsymbol{\xi}[2]}\right]>\\&\sum_{i,j=1}^{N}\pi_i^{[2]}p_{ji}^{[2]}\sum_{\ell=1}^{N}\left[-\gamma_{ij}^{\boldsymbol{\xi}[2,1]}cp_{j\ell}^{[1]}+\gamma_{i\ell}^{\boldsymbol{\xi}[2,1]}bp_{j\ell}^{[1]}+\beta_{ij}^{\boldsymbol{\xi}[2]}\left(r-1\right)+\eta_i^{\boldsymbol{\xi}[2]}\right].\end{aligned}\tag{S67}$$

In Eq. (S67), the $\eta_i^{\boldsymbol{\xi}[2]}$ terms cancel out, leading to

$$\begin{aligned}&\sum_{i,j=1}^{N}\pi_i^{[2]}p_{ji}^{[2]}\sum_{\ell=1}^{N}\left[-\gamma_{ii}^{\boldsymbol{\xi}[2,1]}cp_{i\ell}^{[1]}+\gamma_{i\ell}^{\boldsymbol{\xi}[2,1]}bp_{i\ell}^{[1]}+\beta_{ii}^{\boldsymbol{\xi}[2]}\left(r-1\right)\right]>\\&\sum_{i,j=1}^{N}\pi_i^{[2]}p_{ji}^{[2]}\sum_{\ell=1}^{N}\left[-\gamma_{ij}^{\boldsymbol{\xi}[2,1]}cp_{j\ell}^{[1]}+\gamma_{i\ell}^{\boldsymbol{\xi}[2,1]}bp_{j\ell}^{[1]}+\beta_{ij}^{\boldsymbol{\xi}[2]}\left(r-1\right)\right].\end{aligned}\tag{S68}$$

To compute Eq. (S68), we follow Ref. [1] to obtain $\beta_{ij}^{\boldsymbol{\xi}[2]}$ and $\gamma_{ij}^{\boldsymbol{\xi}[21]}$ as solutions of recursive equations as follows.

For $i=j$, we obtain

$$\begin{aligned}\beta_{ii}^{\boldsymbol{\xi}[2]}&=\xi_i^{[2]}-\widehat{\xi}^{[2]}+\sum_{\left(R^{[1]},\alpha^{[1]}\right)}p^{\circ}_{\left(R^{[2]},\alpha^{[2]}\right)}\beta^{\boldsymbol{\xi}[2]}_{\widetilde{\alpha}^{[2]}(i),\widetilde{\alpha}^{[2]}(i)}\\&=\xi_i^{[2]}-\widehat{\xi}^{[2]}+\frac{1}{N}\sum_{k=1}^{N}p_{ki}^{[2]}\beta_{kk}^{\boldsymbol{\xi}[2]}+\left(1-\frac{1}{N}\sum_{k=1}^{N}p_{ki}^{[2]}\right)\beta_{ii}^{\boldsymbol{\xi}[2]},\end{aligned}\tag{S69}$$

which gives

$$\beta_{ii}^{\boldsymbol{\xi}[2]}=\frac{N\left(\xi_i^{[2]}-\widehat{\xi}_i^{[2]}\right)+\sum_{k=1}^{N}p_{ki}^{[2]}\beta_{kk}^{\boldsymbol{\xi}[2]}}{\sum_{k=1}^{N}p_{ki}^{[2]}}.\tag{S70}$$

For $i\neq j$, we obtain

$$\beta_{ij}^{\boldsymbol{\xi}[2]}=\frac{N\left(\xi_i^{[2]}\xi_j^{[2]}-\widehat{\xi}_i^{[2]}\right)+\sum_{k=1}^{N}p_{ki}^{[2]}\beta_{kj}^{\boldsymbol{\xi}[2]}+\sum_{k=1}^{N}p_{kj}^{[2]}\beta_{ik}^{\boldsymbol{\xi}[2]}}{\sum_{k=1}^{N}p_{ki}^{[2]}+\sum_{k=1}^{N}p_{kj}^{[2]}}.\tag{S71}$$

Finally, we obtain

$$\begin{aligned}
\gamma_{ij}^{\boldsymbol{\xi}[2,1]} &= \xi_i^{[1]}\xi_j^{[2]} - \widehat{\xi}^{[1]}\widehat{\xi}^{[2]} + \sum_{\substack{(R^{[1]},\alpha^{[1]}),\\(R^{[2]},\alpha^{[2]})}} p^{\boldsymbol{\xi}[1]}_{\left(R^{[1]},\alpha^{[1]}\right)} p^{\boldsymbol{\xi}[2]}_{\left(R^{[2]},\alpha^{[2]}\right)} \gamma^{\boldsymbol{\xi}[2,1]}_{\widetilde{\alpha}^{[1]}(i),\widetilde{\alpha}^{[2]}(j)} \\
&= \xi_i^{[1]}\xi_j^{[2]} - \widehat{\xi}^{[1]}\widehat{\xi}^{[2]} + \frac{1}{N^2}\sum_{k_1,k_2=1}^{N} p_{ik_1}^{[1]} p_{k_2 j}^{[2]} \gamma_{k_1 k_2}^{\boldsymbol{\xi}[2,1]} \\
&\quad + \frac{1}{N}\left(1-\frac{1}{N}\right)\sum_{k_1=1}^{N} p_{ik_1}^{[1]}\gamma_{k_1 j}^{\boldsymbol{\xi}[2,1]} \\
&\quad + \frac{1}{N}\left(1-\frac{1}{N}\sum_{k_2=1}^{N} p_{k_2 j}^{[2]}\right)\sum_{k_2=1}^{N} p_{k_2 j}^{[2]}\gamma_{ik_2}^{\boldsymbol{\xi}[2,1]} \\
&\quad + \left(1-\frac{1}{N}\right)\left(1-\frac{1}{N}\sum_{k_2=1}^{N} p_{k_2 j}^{[2]}\right)\gamma_{ij}^{\boldsymbol{\xi}[2,1]},
\end{aligned} \tag{S72}$$

which gives

$$\gamma_{ij}^{\xi[21]} = \frac{N^2\left(\xi_i^{[1]}\xi_j^{[2]} - \xi_i^{[1]}\xi_j^{[2]}\right) + \sum_{k_1,k_2=1}^{N} p_{ik_1}^{[1]} p_{k_2 j}^{[2]}\gamma_{k_1k_2}^{\boldsymbol{\xi}[2,1]} + \left(N - \sum_{k_2=1}^{N} p_{k_2 j}^{[2]}\right)\sum_{k_1=1}^{N} p_{ik_1}^{[1]}\gamma_{k_1 j}^{\boldsymbol{\xi}[2,1]} + (N-1)\sum_{k_2=1}^{N} p_{k_2 j}^{[2]}\gamma_{ik_2}^{\boldsymbol{\xi}[2,1]}}{N + N\sum_{k_2=1}^{N} p_{k_2 j}^{[2]} - \sum_{k_2=1}^{N} p_{k_2 j}^{[2]}}. \tag{S73}$$

S4 Effects of the network structure and initial condition in layer 1 on $\frac{\partial r^*}{\partial b}$ and $\frac{\partial r^*}{\partial c}$

To explore reasons for striped patterns in Fig. 3 in the main text, we computed the distribution of $\left(\frac{\partial r^*}{\partial b}, \frac{\partial r^*}{\partial c}\right)$ for arbitrarily selected four layer-1 networks with $N = 6$ individuals. For each layer-1 network, we first fixed an initial condition in layer 1, i.e., the sole replica node initially hosting a cooperator. Then, for all combinations of one of the four arbitrarily selected layer-2 network, its isomorphism, and initial condition (i.e., the sole replica node in layer 2 that initially hosts the mutant), we computed $\left(\frac{\partial r^*}{\partial b}, \frac{\partial r^*}{\partial c}\right)$. We avoided using all possible networks as the layer-2 network because doing so would make scattergrams too crowded.

We show $\left(\frac{\partial r^*}{\partial b}, \frac{\partial r^*}{\partial c}\right)$ for each pair of the layer-1 network and its initial condition in Fig. S1. Each filled dot represents the result for a pair of the layer-2 network and its initial condition. The first six panels show the results for one arbitrarily selected layer-1 network. Each of the six panels corresponds to an initial condition in layer 1. We visualize the layer-1 network and its initial condition within each panel; we recall that the black and orange circles represent the resident and mutant replica nodes, respectively. The next six panels show the results for a second layer-1 network, and so forth. Some panels are blank because the corresponding initial condition is the same as one that appeared in an earlier panel due to the symmetry of the network. For example, in the top row of Fig. S1, nodes 1 and 2 are structurally equivalent. Therefore, placing the initial cooperator on node 1 versus node 2 produces exactly the same result, which is why the second panel does not show any dots.

We find that different layer-1 networks and their initial conditions produce different sets of stripes of the data points in general. Some stripes are only present in some of the panels. Therefore, we conclude that the network structure and initial condition in layer 1 are partial determinants of the stripe patterns observed in Fig. 3 in the main text.

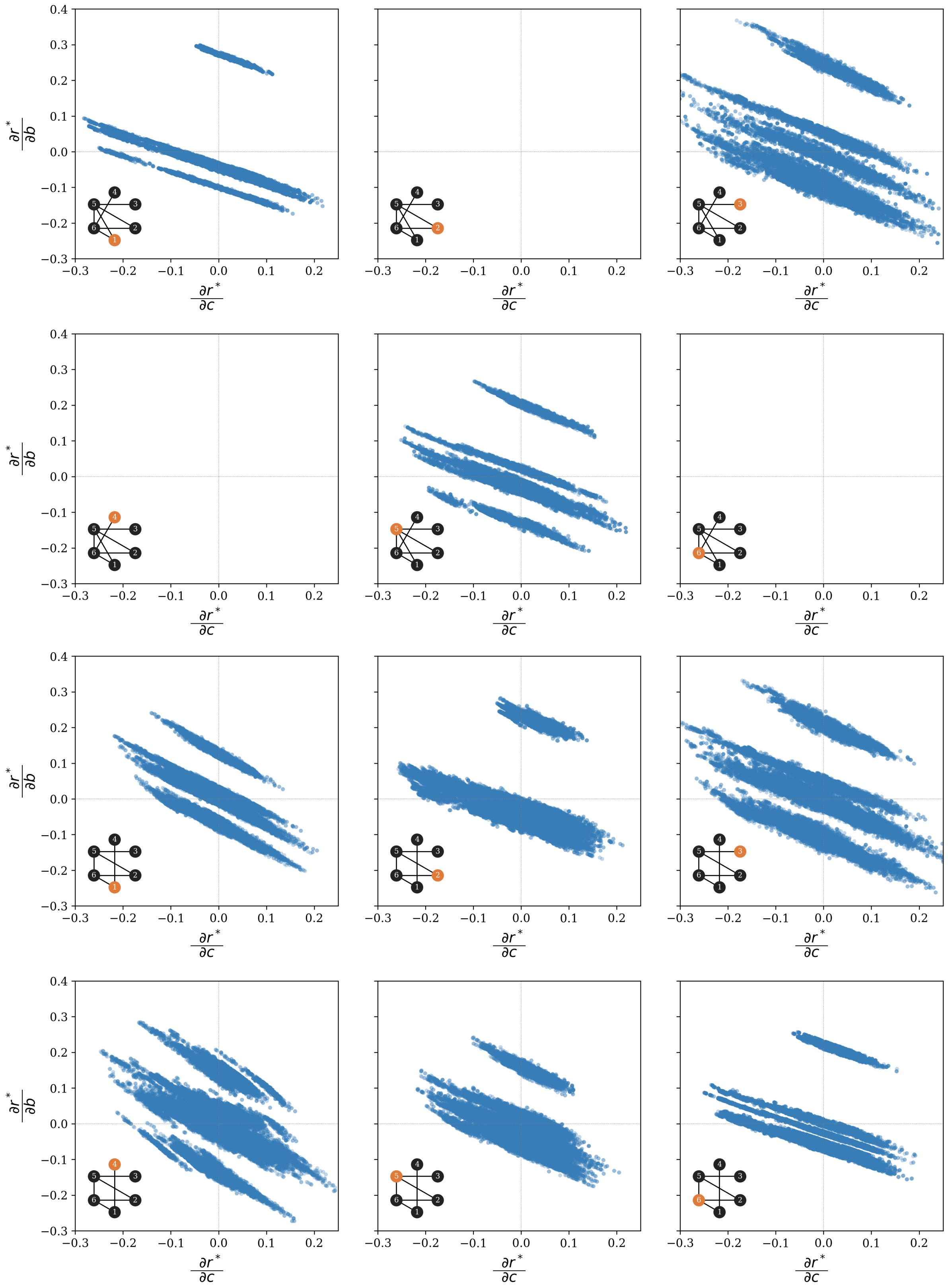

Figure S1: Distribution of $\frac{\partial r^*}{\partial b}$ and $\frac{\partial r^*}{\partial c}$ over layer-2 networks and their initial conditions. The visualization of the network within each panel shows the layer-1 network and its initial condition used in the panel. Each filled dot represents a pair of the layer-2 network and its initial condition. Dots are not present in four of the 24 panels because these four cases are the same as a pair of the layer-1 network and its initial condition that appears in an earlier panel.

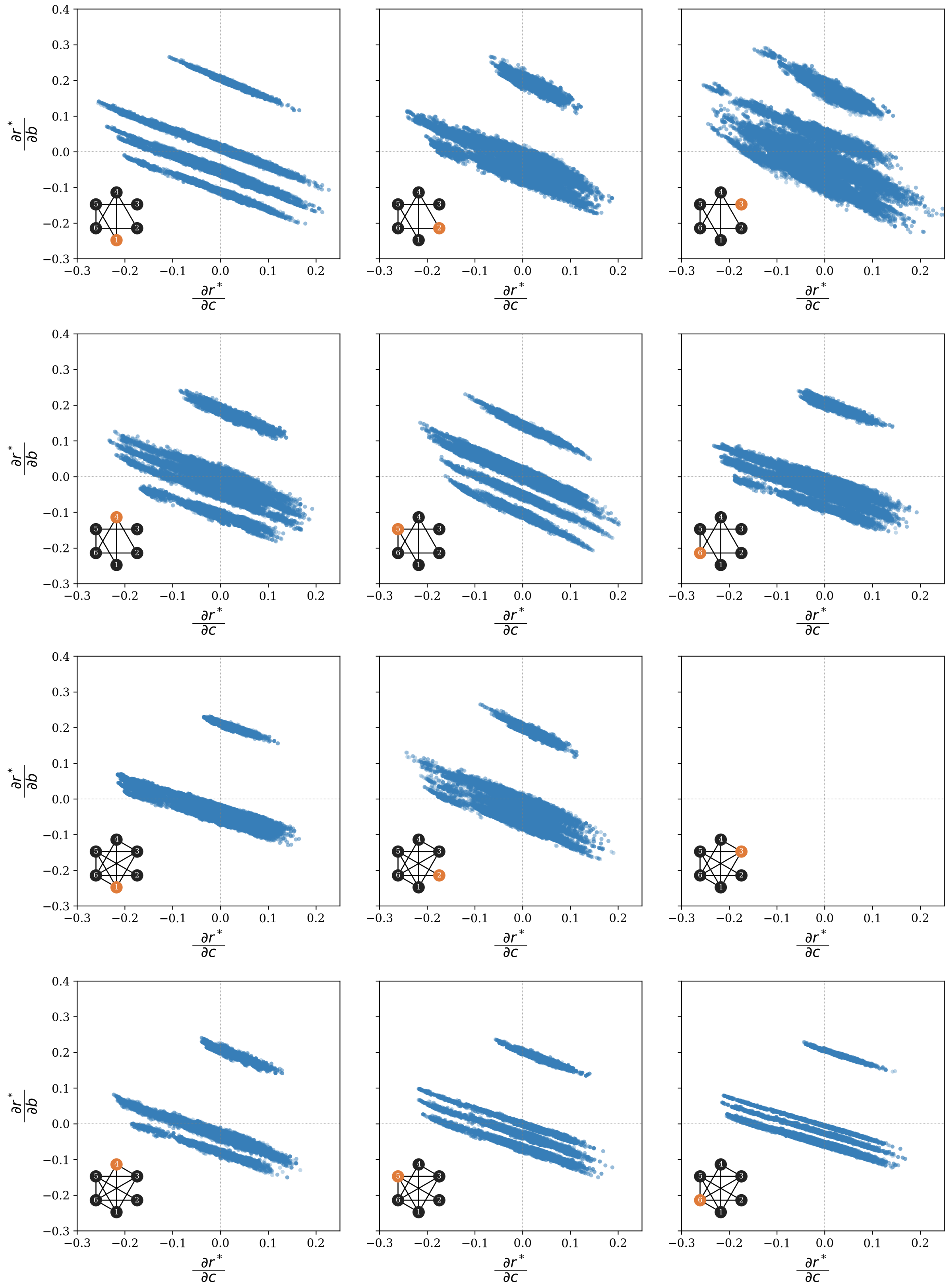

Figure S1: (Continued.)

S5 Evolution of the cooperator and mutant on the coupled ring network

We examine the coupled ring network depicted in Fig. 4(a) in the main text under the dB-dB rule. The expressions for $\theta_i^{\boldsymbol{\xi}[1]}$ and $\phi_{m,n}^{\boldsymbol{\xi}[1,2]}$ were derived in Ref. [1]. By following their work, we set $\xi_1^{[1]} = 1$, $\xi_j^{[1]} = 0$ for $j \neq 1$, $\xi_i^{[2]} = 1$, and $\xi_j^{[2]} = 0$ for $j \neq i$. Due to the symmetry of the ring network, this setting covers all possible initial conditions although we assumed $i = 1$ in Fig. 4(a) for demonstration. Let d denote the distance between the node with the initial cooperator in layer 1 and that with the initial mutant in layer 2 when we superpose the two identical rings, one from each layer, to regard the superposition as a single ring network. We obtain

$$\theta_1^{\boldsymbol{\xi}[1]} = -\frac{N-1}{2}, \tag{S74}$$

$$\theta_2^{\boldsymbol{\xi}[1]} = -\frac{N-2}{2}, \tag{S75}$$

$$\theta_3^{\boldsymbol{\xi}[1]} = -\frac{3(N-2)}{4}, \tag{S76}$$

$$\phi_{0,1}^{\boldsymbol{\xi}[1,2]} = -\sum_{\ell=1}^{N-1} \frac{\cos\left(\frac{2\pi\ell d}{N}\right)}{2N-1+\cos\left(\frac{2\pi\ell}{N}\right)}, \tag{S77}$$

$$\phi_{2,0}^{\boldsymbol{\xi}[1,2]} = -2(N-1)\phi_{0,1}^{\boldsymbol{\xi}[1,2]} - N\delta_{d,0} + 1, \tag{S78}$$

$$\phi_{2,1}^{\boldsymbol{\xi}[1,2]} = (4N^2 - 6N + 3)\phi_{0,1}^{\boldsymbol{\xi}[1,2]} - \frac{N}{2}\delta_{d,1} + 2N(N-1)\delta_{d,0} - 2N + 3. \tag{S79}$$

By substituting Eqs. (S74)–(S79) in Eq. (3) in the main text, we obtain the condition under which selection favors the cooperator in layer 1 as follows:

$$\begin{aligned} 0 &< c\theta_2^{\boldsymbol{\xi}[1]} + b\left(\theta_1^{\boldsymbol{\xi}[1]} - \theta_3^{\boldsymbol{\xi}[1]}\right) - (r-1)\phi_{2,0}^{\boldsymbol{\xi}[1,2]} \\ &= \frac{b}{4}(N-4) - \frac{c}{2}(N-2) + (r-1)\left[2(N-1)\phi_{0,1}^{\boldsymbol{\xi}[1,2]} + N\delta_{d,0} - 1\right], \end{aligned} \tag{S80}$$

which we can rearrange to

$$\frac{b}{c} > \left(\frac{b}{c}\right)^* \equiv \frac{(N-2)}{2(N-4)} - \frac{4(r-1)}{(N-4)c}\left[2(N-1)\phi_{0,1}^{\boldsymbol{\xi}[1,2]} + N\delta_{d,0} - 1\right] \tag{S81}$$

assuming $N \geq 4$. Equation (S81) suggests that $(b/c)^*$ for the coupled ring network can be moved from the value for the one-layer ring, $(N-2)/[2(N-4)]$, by choosing $r \neq 1$. Equation (S77) leads to

$$\lim_{N\to\infty} \phi_{0,1}^{\boldsymbol{\xi}[1,2]} = -\int_0^1 \frac{\cos(2\pi d x)}{2}\mathrm{d}x = 0. \tag{S82}$$

By combining Eqs. (S81) and (S82), we obtain

$$\lim_{N\to\infty}\left(\frac{b}{c}\right)^* = \begin{cases} \frac{1}{2} & \text{if } d > 0, \\ \frac{1}{2} - \frac{4(r-1)}{c} & \text{if } d = 0. \end{cases} \tag{S83}$$

Therefore, on large coupled ring networks, the coupling with the constant-selection layer considerably impacts the emergence of cooperation if and only if the initial cooperator and mutant are on the same individual.

Similarly, by substituting Eqs. (S74)–(S79) in Eq. (4) in the main text, we obtain the condition under which selection favors the mutant in layer 2 as follows:

$$\begin{aligned}0 &< -(r-1)\theta_2^{\boldsymbol{\xi}[1]} + c\,\phi_{2,0}^{\boldsymbol{\xi}[1,2]} + b\Big(\phi_{0,1}^{\boldsymbol{\xi}[1,2]} - \phi_{2,1}^{\boldsymbol{\xi}[1,2]}\Big) \\ &= \frac{N-2}{2}(r-1) - 2(N-1)\left[b(2N-1)+c\right]\phi_{0,1}^{\boldsymbol{\xi}[1,2]} - N\left[2b(N-1)+c\right]\delta_{d,0} + \frac{bN}{2}\,\delta_{d,1} + c + b(2N-3),\end{aligned} \tag{S84}$$

which is equivalent to

$$r > 1 + \frac{4(N-1)}{N-2}\left[b(2N-1)+c\right]\phi_{0,1}^{\boldsymbol{\xi}[1,2]} + \frac{2N}{N-2}\left[2b(N-1)+c\right]\delta_{d,0} - \frac{bN}{N-2}\,\delta_{d,1} - \frac{c}{N-2} + \frac{b(2N-3)}{N-2}. \tag{S85}$$

S6 Evolution of the cooperator and mutant on the coupled complete graph

In this section, we derive the condition for favoring the cooperator and mutant when both layers are the complete graph with N nodes. We assume the unweighted complete graphs without self-loops. We also assume the initial condition used in Fig. 4(c) in the main text, in which the individual cooperating in layer 1 coincides with the mutant in layer 2.

S6.A Derivation of $\theta_n^{\boldsymbol{\xi}[1]}$

We first compute

$$\theta_n^{\boldsymbol{\xi}[1]} = \sum_{i=1}^{N}\sum_{j=1}^{N} \pi_i^{[1]} \left(p^{[1]}\right)_{ij}^{(n)} \beta_{ij}^{\boldsymbol{\xi}[1]}, \tag{S86}$$

for the complete graph. The one-step transition probability matrix of the random walk, $P = \left(p_{ij}^{[1]}\right)$, is given by

$$P = \frac{1}{N-1}(J - I), \tag{S87}$$

where J is matrix with all its entries being 1, and I is the identity matrix. Matrix P has eigenvalues 1 and $\lambda := -\frac{1}{N-1}$ with multiplicities 1 and $N-1$, respectively. The stationary density of the random walk is given by

$$\pi_i^{[1]} = \frac{1}{N}, \quad i \in \{1, \dots, N\}. \tag{S88}$$

Using $J^2 = NJ$ and the fact that J and I commute, we obtain

$$\begin{aligned} P^n =& \frac{1}{(N-1)^n} \sum_{k=0}^{n} \binom{n}{k} J^k (-I)^{n-k} \\ =& \left(-\frac{1}{N-1}\right)^n I + \frac{J}{(N-1)^n} \sum_{k=1}^{n} \binom{n}{k} N^{k-1} (-1)^{n-k} \\ =& \frac{1}{N} J + \lambda^n \left(I - \frac{J}{N}\right), \end{aligned} \tag{S89}$$

where $\binom{n}{k}$ is the binomial coefficient. By substituting Eqs. (S88) and (S89) in Eq. (S86), we obtain

$$\begin{aligned} \theta_n^{\boldsymbol{\xi}[1]} =& \frac{1}{N} \sum_{i,j=1}^{N} (P^n)_{ij}\, \beta_{ij}^{\boldsymbol{\xi}[1]} \\ =& \frac{1}{N} \sum_{i,j=1}^{N} \left[\frac{1}{N} J_{ij} + \lambda^n \left(\delta_{ij} - \frac{1}{N} J_{ij}\right)\right] \beta_{ij}^{\boldsymbol{\xi}[1]} \\ =& \frac{1}{N^2} \sum_{i,j=1}^{N} J_{ij} \beta_{ij}^{\boldsymbol{\xi}[1]} + \frac{\lambda^n}{N} \left(\sum_{i=1}^{N} \beta_{ii}^{\boldsymbol{\xi}[1]} - \sum_{i,j=1}^{N} \frac{1}{N} J_{ij} \beta_{ij}^{\boldsymbol{\xi}[1]}\right) \\ =& \frac{1}{N^2} \sum_{i,j=1}^{N} \beta_{ij}^{\boldsymbol{\xi}[1]} + \frac{\lambda^n}{N} \left(\sum_{i=1}^{N} \beta_{ii}^{\boldsymbol{\xi}[1]} - \sum_{i,j=1}^{N} \frac{1}{N} \beta_{ij}^{\boldsymbol{\xi}[1]}\right). \end{aligned} \tag{S90}$$

To simplify Eq. (S90), we note that substitution of $\pi_i = \frac{1}{N}, \forall i$ in Eq. (S37) yields $\sum_{i=1}^{N} \beta_{ii}^{\boldsymbol{\xi}[1]} = 0$. By substituting this equality in Eq. (S90), we obtain

$$\begin{aligned}
\theta_n^{\boldsymbol{\xi}[1]} =& \frac{1}{N^2} \sum_{i,j=1}^{N} \beta_{ij}^{\boldsymbol{\xi}[1]} + \frac{\lambda^n}{N} \left(\sum_{i=1}^{N} \beta_{ii}^{\boldsymbol{\xi}[1]} - \sum_{i,j=1}^{N} \frac{1}{N} \beta_{ij}^{\boldsymbol{\xi}[1]} \right) \\
=& \frac{1}{N^2} \sum_{i,j=1;i\neq j}^{N} \beta_{ij}^{\boldsymbol{\xi}[1]} + \frac{\lambda^n}{N} \left(0 - \sum_{i,j=1;i\neq j}^{N} \frac{1}{N} \beta_{ij}^{\boldsymbol{\xi}[1]} \right) \\
=& \frac{1-\lambda^n}{N^2} \sum_{i,j=1;i\neq j}^{N} \beta_{ij}^{\boldsymbol{\xi}[1]} \\
\equiv& \frac{1-\lambda^n}{N^2} S_{\text{off}}.
\end{aligned} \tag{S91}$$

We need to obtain S_{off} from Eq. (S54). To simplify Eq. (S54) for the complete graph, we first note that variables $\xi_i^{[1]}$ and $\xi_j^{[1]}$ are 0 or 1 depending on whether a defector or cooperator, respectively, occupies the node. Because we have only one cooperator in our initial condition, both $\xi_i^{[1]}$ and $\xi_j^{[1]}$, where $i \neq j$, cannot be 1, leading to $\xi_i^{[1]} \xi_j^{[1]} = 0$. The weighted reproductive value $\widehat{\xi}^{[1]} = 1/N$ comes from the definition of the RV-weighted frequency, Eq. (S4), and Eq. (S5). Because all the nodes in the complete graph have the same reproductive value due to symmetry and only one node has a cooperator, the RV-weighted frequency is equal to $1/N$. Therefore, we obtain

$$\xi_i^{[1]} \xi_j^{[1]} - \hat{\xi}^{[1]} = -\frac{1}{N}. \tag{S92}$$

By substituting Eq. (S92), $p_{ik}^{[1]} = 1/(N-1)$ for $k \neq i$, and $p_{ik}^{[1]} = 0$ for $k = i$ in Eq. (S54), we obtain

$$\beta_{ij}^{\boldsymbol{\xi}[1]} = -\frac{1}{2} + \frac{1}{2} \sum_{k=1;k\neq i}^{N} \frac{\beta_{kj}^{\boldsymbol{\xi}[1]}}{N-1} + \frac{1}{2} \sum_{k=1;k\neq j}^{N} \frac{\beta_{ik}^{\boldsymbol{\xi}[1]}}{N-1}, \qquad (i \neq j). \tag{S93}$$

By summing both sides of Eq. (S93) over the i and j values with $i \neq j$, we obtain

$$S_{\text{off}} = -\frac{N(N-1)}{2} + \frac{1}{2} \frac{N-2}{N-1} S_{\text{off}} + \frac{1}{2} \frac{N-2}{N-1} S_{\text{off}}. \tag{S94}$$

Equation (S94) leads to

$$S_{\text{off}} = -\frac{N(N-1)^2}{2}. \tag{S95}$$

Substitution Eq. (S95) in Eq. (S91) yields

$$\theta_n^{\boldsymbol{\xi}[1]} = -\frac{(N-1)^2}{2N} \left[1 - \left(-\frac{1}{N-1} \right)^n \right], \tag{S96}$$

where $n \in \{1, 2, \ldots\}$.

S6.B Derivation of $\phi_{n,m}^{\boldsymbol{\xi}[1,2]}$

We now compute

$$\phi_{n,m}^{\boldsymbol{\xi}[1,2]} := \sum_{i,j=1}^{N} \pi_i^{[1]} \left(p^{[1,2]} \right)_{ij}^{(n,m)} \gamma_{ij}^{\boldsymbol{\xi}[1,2]}. \tag{S97}$$

Because the two layers are both complete graph on N nodes, we obtain

$$\left(p^{[1,2]}\right)^{(n,m)} = P^n P^m = P^{n+m} \tag{S98}$$

and

$$\phi_{n,m}^{\boldsymbol{\xi}[1,2]} = \sum_{i,j=1}^{N} \pi_i^{[1]} \left(p^{[1,2]}\right)_{ij}^{(n,m)} \gamma_{ij}^{\boldsymbol{\xi}[1,2]} = \frac{1}{N}\sum_{i,j}(P^{n+m})_{ij}\, \gamma_{ij}^{\boldsymbol{\xi}[1,2]}, \tag{S99}$$

where we used $\pi_i^{[1]} = 1/N$.

To compute $\gamma_{ij}^{\boldsymbol{\xi}[1,2]}$, which we write γ_{ij} in the remainder of this section for notational simplicity, we set with $\xi_1^{[1]} = \xi_1^{[2]} = 1$ and $\xi_j^{[L]} = 0$ for $j \neq 1$, $L \in \{1,2\}$. This assumption corresponds to the initial condition that we have assumed, in which the sole initial cooperator in layer 1 is the sole initial mutant in layer 2. We obtain $\widehat{\xi}^{[1]} = \widehat{\xi}^{[2]} = 1/N$ and $\vec{\xi}^{[1]}\vec{\xi}^{[2]\top} - \widehat{\xi}^{[1]}\widehat{\xi}^{[2]}J = U - \frac{1}{N^2}J$, where $\vec{\xi}^{[L]} = (\xi_1^{[L]}, \ldots, \xi_i^{[L]}, \ldots, \xi_N^{[L]})^\top$, $U := \boldsymbol{e}_1\boldsymbol{e}_1^\top$, and vector $\boldsymbol{e}_1$ is the standard basis vector, i.e., $\boldsymbol{e}_1 = (1, 0, \ldots, 0)^\top$. The expression of γ_{ij} with $i \neq j$, shown in Eq. (S56), simplifies to

$$\gamma_{ij} = \frac{N^2}{2N-1}\left(U_{ij} - \frac{1}{N^2}\right) + \frac{1}{2N-1}(P\gamma P)_{ij} + \frac{N-1}{2N-1}\left((P\gamma)_{ij} + (\gamma P)_{ij}\right). \tag{S100}$$

We also note that $\sum_{i=1}^N \pi_i \gamma_{ii} = 0$ simplifies to

$$\frac{1}{N}\sum_{i=1}^{N}\gamma_{ii} = 0. \tag{S101}$$

Using Eqs. (S89), (S99), and (S101), we obtain

$$\begin{aligned}
\phi_{n,m} &= \frac{1}{N}\left[\sum_{i=1}^{N}\left(\frac{1}{N} + \frac{N-1}{N}\lambda^{n+m}\right)\gamma_{ii} + \sum_{i,j=1;i\neq j}^{N}\left(\frac{1}{N} - \frac{1}{N}\lambda^{n+m}\right)\gamma_{ij}\right] \\
&= \frac{1-\lambda^{n+m}}{N^2}\sum_{i,j=1;i\neq j}^{N}\gamma_{ij} \\
&\equiv \frac{1-\lambda^{n+m}}{N^2}\overline{S}_{\text{off}}.
\end{aligned} \tag{S102}$$

To calculate $\overline{S}_{\text{off}}$, we sum Eq. (S100) over all i and j with $i \neq j$. The sum of the first term on the right-hand side of Eq. (S100) becomes

$$\sum_{i,j=1;i\neq j}^{N}\frac{N^2}{2N-1}\left(U_{ij} - \frac{1}{N^2}\right) = \frac{N^2}{2N-1}\left[0 - \frac{N(N-1)}{N^2}\right] = -\frac{N(N-1)}{2N-1}. \tag{S103}$$

The sum of the second term on the right-hand side of Eq. (S100) is written as

$$\sum_{i,j=1;i\neq j}^{N}(P\gamma P)_{ij} = \sum_{k,\ell=1}^{N}\gamma_{k\ell}\,T(k,\ell), \tag{S104}$$

where

$$T(k,\ell) := \sum_{i,j=1;i\neq j}^{N} p_{ik}p_{j\ell}. \tag{S105}$$

Because

$$p_{ik} = \begin{cases} 0 & \text{if } i = k, \\ \frac{1}{N-1} & \text{if } i \neq k, \end{cases} \tag{S106}$$

$T(k, \ell)$ counts ordered pairs (i, j) with $i \neq j$, $i \neq k$, $j \neq \ell$ with weight $(N-1)^{-2}$. Among the $N(N-1)$ pairs of (i, j) with $i \neq j$, there are $2(N-1)$ pairs of (i, j) satisfying $i = k$ or $j = \ell$, if $k = \ell$. If $k \neq \ell$, there are $2(N-1) - 1$ such (i, j) pairs. Therefore, we obtain

$$\#\{(i,j)\} = \begin{cases} (N-2)(N-1) & \text{if } k = \ell, \\ (N-2)(N-1) + 1 & \text{if } k \neq \ell, \end{cases} \tag{S107}$$

where $\#\{(i,j)\}$ is the number of (i, j) pairs counted in $T(k, \ell)$. Because each (i, j) pair contributes weight $(N-1)^{-2}$, we obtain

$$T(k,\ell) = \begin{cases} \dfrac{N-2}{N-1} & \text{if } k = \ell, \\ \dfrac{N^2 - 3N + 3}{(N-1)^2} & \text{if } k \neq \ell. \end{cases} \tag{S108}$$

By substituting Eq. (S108) in Eq. (S104) and using Eq. (S101), we obtain

$$\sum_{i,j=1; i \neq j}^{N} (P \gamma P)_{ij} = \frac{N^2 - 3N + 3}{(N-1)^2} \overline{S}_{\text{off}}. \tag{S109}$$

To calculate the sum of the third term on the right-hand side of Eq. (S100), we first obtain

$$\begin{aligned} \sum_{i,j=1; i \neq j}^{N} (P\gamma)_{ij} &= \sum_{i,j=1; i \neq j}^{N} \sum_{k=1}^{N} p_{ik} \gamma_{kj} \\ &= \sum_{k,j=1}^{N} \gamma_{kj} \sum_{i=1; i \neq j}^{N} p_{ik} \\ &= \sum_{k,j=1}^{N} \gamma_{kj} \left(1 - p_{jk}\right) \\ &= \overline{S}_{\text{off}} - \sum_{k,j=1}^{N} p_{jk} \gamma_{kj} \\ &= \overline{S}_{\text{off}} - \frac{\overline{S}_{\text{off}}}{N-1} \\ &= \frac{N-2}{N-1} \overline{S}_{\text{off}}. \end{aligned} \tag{S110}$$

We used Eq. (S101) to derive the third last equality. We used Eqs. (S101) and (S106) to derive the second last equality. By symmetry, the same holds true for $\sum_{i \neq j} (\gamma P)_{ij}$. Therefore, we obtain

$$\sum_{i,j=1; i \neq j}^{N} \left((P\gamma)_{ij} + (\gamma P)_{ij} \right) = \frac{2(N-2)}{N-1} \overline{S}_{\text{off}}. \tag{S111}$$

By summing Eq. (S100) over all $i, j \in \{1, \ldots, N\}$ with $i \neq j$ and using Eqs. (S103), (S109), and (S111), we obtain

$$\overline{S}_{\text{off}} = -\frac{N(N-1)}{2N-1} + \frac{1}{2N-1} \cdot \frac{N^2 - 3N + 3}{(N-1)^2} \overline{S}_{\text{off}} + \frac{N-1}{2N-1} \cdot \frac{2(N-2)}{N-1} \overline{S}_{\text{off}}. \tag{S112}$$

Equation (S112) leads to

$$\overline{S}_{\text{off}} = -\frac{(N-1)^3}{2N-3}. \tag{S113}$$

By substituting Eq. (S113) and $\lambda = -1/(N-1)$ in Eq. (S102), we obtain

$$\phi_{n,m} = -\frac{(N-1)^3}{N^2(2N-3)}\left[1-\left(-\frac{1}{N-1}\right)^{n+m}\right]. \tag{S114}$$

S6.C Condition for favoring the cooperator and mutant

By substituting Eqs. (S96) and (S114) in Eq. (3) in the main text, we find that spite is favored on the coupled complete graph if

$$\frac{b}{c} < \left(\frac{b}{c}\right)^* = -(N-1) - \frac{r-1}{c}\cdot\frac{2(N-1)^2}{N(2N-3)}. \tag{S115}$$

When N is large, the first term on the right-hand of Eq. (S115) dominates the second term such that the coupling the two complete graphs does not alter the condition for spite substantially. However, the effect is large when N is small.

The condition for the selection of the mutant, Eq. (4) in the main text, reads

$$A = -\frac{2(N-1)}{N(2N-3)}\left(\frac{b}{N-1}+c\right). \tag{S116}$$

This result implies that r^* is substantially different from 1 if and only if N is small. We also find that the effect of c on r^* is $N-1$ times larger than that of b.

S7 Evolution of the the cooperator and mutant on the coupled star graph

We derive the condition for favoring the cooperator and mutant in two-layer star graphs under the dB-dB rule. See Fig. S2 for the network; we focus on this particular two-layer star graph and initial condition with varying N, as in the previous study [1] (see SI Fig. 16 of their paper). The expressions for $\theta_i^{\boldsymbol{\xi}[1]}$ and $\phi_{m,n}^{\boldsymbol{\xi}[1,2]}$ were derived [1] as follows:

$$\theta_1^{\boldsymbol{\xi}[1]} = -\frac{2N-3}{2(N-1)}, \tag{S117}$$

$$\theta_2^{\boldsymbol{\xi}[1]} = -\frac{3(N-2)}{4(N-1)}, \tag{S118}$$

$$\theta_3^{\boldsymbol{\xi}[1]} = -\frac{2N-3}{2(N-1)}, \tag{S119}$$

$$\phi_{0,1}^{\boldsymbol{\xi}[1,2]} = -\frac{N\left(8N^5-52N^4+112N^3-107N^2+46N-8\right)}{8(2N-1)(3N-2)(N-1)^4}, \tag{S120}$$

$$\phi_{2,0}^{\boldsymbol{\xi}[1,2]} = -\frac{N^2(N-2)}{2(2N-1)(3N-2)(N-1)^2}, \tag{S121}$$

$$\phi_{2,1}^{\boldsymbol{\xi}[1,2]} = -\frac{N\left(8N^5-44N^4+84N^3-79N^2+38N-8\right)}{8(2N-1)(3N-2)(N-1)^4}. \tag{S122}$$

Note that $\theta_1^{\boldsymbol{\xi}[1]} = \theta_3^{\boldsymbol{\xi}[1]}$ because the star graph is a special case of the complete bipartite graph. By substituting Eqs. (S117)–(S122) in Eq. (3) in the main text, we obtain the condition under which selection favors the cooperator in layer 1 as follows:

$$\begin{aligned}
0 &< c\,\theta_2^{\boldsymbol{\xi}[1]} + b\left(\theta_1^{\boldsymbol{\xi}[1]} - \theta_3^{\boldsymbol{\xi}[1]}\right) - (r-1)\,\phi_{2,0}^{\boldsymbol{\xi}[1,2]} \\
&= -\frac{3c(N-2)}{4(N-1)} + \frac{N^2(N-2)(r-1)}{2(2N-1)(3N-2)(N-1)^2} \\
&= \frac{(N-2)\left[2N^2(r-1) - 3c\,(N-1)(2N-1)(3N-2)\right]}{4(N-1)^2(2N-1)(3N-2)}.
\end{aligned} \tag{S123}$$

For $N > 2$, Eq. (S123) is equivalent to

$$2N^2(r-1) - 3c\,(N-1)(2N-1)(3N-2) > 0. \tag{S124}$$

Remarkably, the condition does not depend on b.

Similarly, by substituting Eqs. (S117)–(S122) in Eq. (4) in the main text, we obtain the condition under which selection favors the mutant in layer 2 as follows:

$$\begin{aligned}
0 &< -(r-1)\,\theta_2^{\boldsymbol{\xi}[1]} + c\,\phi_{2,0}^{\boldsymbol{\xi}[1,2]} + b\left(\phi_{0,1}^{\boldsymbol{\xi}[1,2]} - \phi_{2,1}^{\boldsymbol{\xi}[1,2]}\right) \\
&= \frac{3(N-2)(r-1)}{4(N-1)} - c\left[\frac{N^2(N-2)}{2(2N-1)(3N-2)(N-1)^2}\right] \\
&\quad + b\left[-\frac{N\left(8N^5-52N^4+112N^3-107N^2+46N-8\right)}{8(2N-1)(3N-2)(N-1)^4}\right. \\
&\quad \left. + \frac{N\left(8N^5-44N^4+84N^3-79N^2+38N-8\right)}{8(2N-1)(3N-2)(N-1)^4}\right] \\
&= \frac{(N-2)\left\{2N^2\left[b(2N-1)-c(N-1)\right] + 3(N-1)^2(2N-1)(3N-2)(r-1)\right\}}{4(N-1)^3(2N-1)(3N-2)}.
\end{aligned} \tag{S125}$$

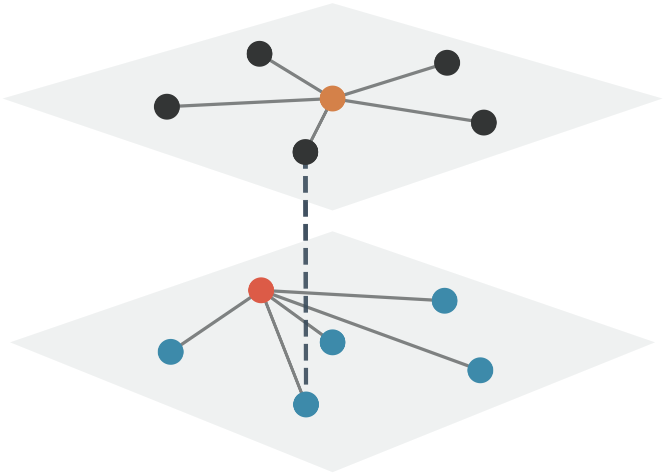

Figure S2: Two-layer star graph and the initial condition that we analyze. Note that the hub node in the first layer is not the hub node in layer 2. The node colors are the same as those in Fig. 1 in the main text; orange represents the cooperator in layer 1, black represents the defector layer 1, blue represents the resident in layer 2, and red represents the mutant in layer 2. The two replica nodes connected by the dashed line represent the same individual, shown as an example.

For $N > 2$, Eq. (S125) is equivalent to

$$r > 1 - \frac{2N^2\left[b(2N-1) - c(N-1)\right]}{3(N-1)^2(2N-1)(3N-2)}. \tag{S126}$$

S8 Mean and standard deviation of $\left|\frac{\mathbf{d}(b/c)^*}{\mathbf{d}r}\right|$ for two-layer ER and BA networks with $N = 15$ individuals

We showed in Fig. 5 in the main text the median and the 5th and 95th percentiles of $|\mathrm{d}(b/c)^*/\mathrm{d}r|$ for two-layer ER and BA networks with $N = 15$ individuals under the dB-dB and dB-Bd updating rules. Figure S3 shows the mean and standard deviation of $|\mathrm{d}(b/c)^*/\mathrm{d}r|$ in the corresponding cases. We find that, while the mean does not behave monotonically with respect to the network density parameters (i.e., p_1, p_2, $\overline{m}_1$, and $\overline{m}_2$), the main trend that $|\mathrm{d}(b/c)^*/\mathrm{d}r|$ increases as the density of edges in layer 1 (i.e., p_1 or $\overline{m}_1$) increases remains the same as in Fig. 5.

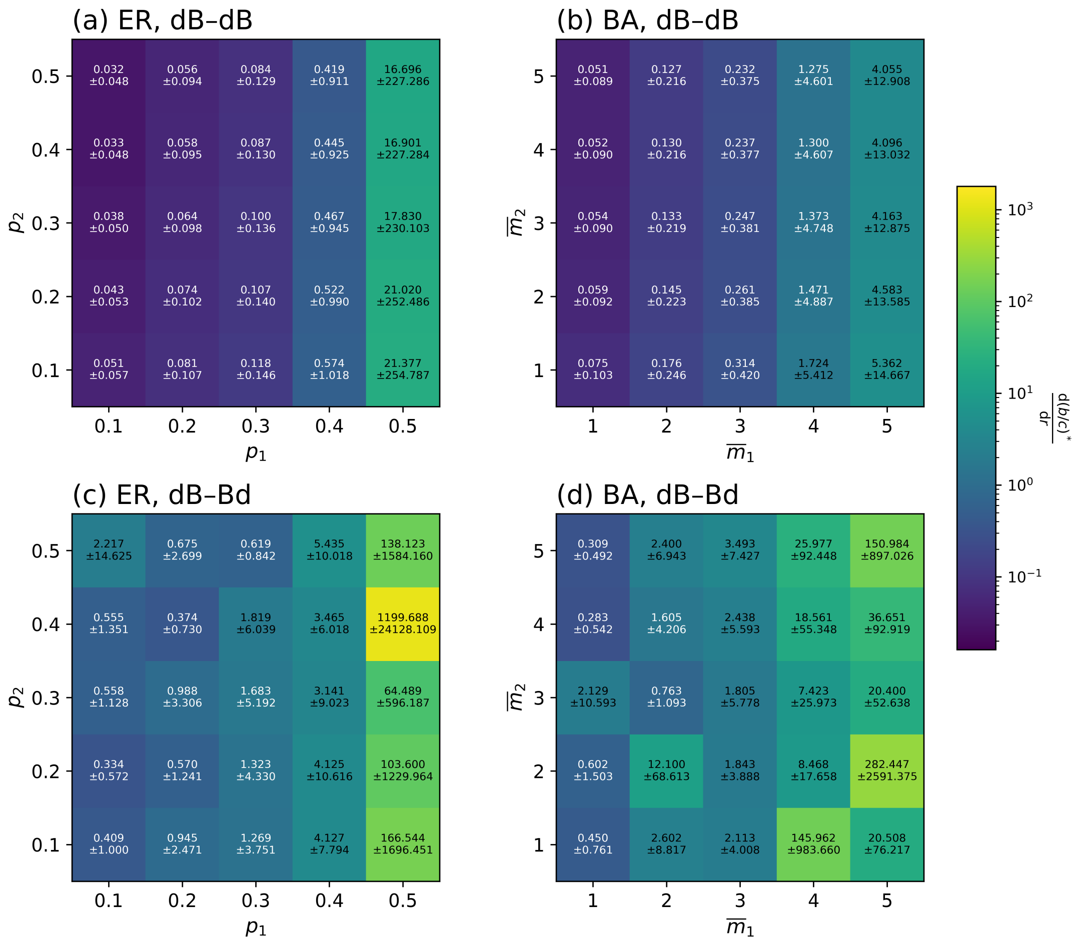

Figure S3: Mean, μ, and standard deviation, σ, of $|\mathrm{d}(b/c)^*/\mathrm{d}r|$ for two-layer ER and BA networks with $N = 15$ individuals. (a) ER, dB-dB rule. (b) BA, dB-dB rule. (c) ER, dB-Bd rule. (d) BA, dB-Bd rule. For each (p_1, p_2) or $(\overline{m}_1, \overline{m}_2)$, we computed μ and σ on the basis of all the pairs of two-layer network and initial condition for which cooperation can be favored (i.e., $(b/c)^* > 0$ for the layer-1 network).

S9 "Fixed benefits, fixed costs" goods scheme

We analyze the case of the ff goods scheme [9]. We first derive the conditions under which the cooperator and mutant are favored under the ff goods scheme. Then, we numerically investigate the conditions for the two-layer networks used in the main text.

S9.A Computation of $(b/c)^*$ and r^*

We recall that, under the pf goods analyzed in the main text, the payoff of the ith individual from layer 1 is given by

$$u_i^{[1]} = -cx_i^{[1]} + \sum_{j=1}^{N} b \frac{w_{ij}^{[1]}}{s_i^{[1]}} x_j^{[1]}. \tag{S127}$$

Under the ff goods, it changes to

$$u_i^{[1]} = -cx_i^{[1]} + \sum_{j=1}^{N} b \frac{w_{ij}^{[1]}}{s_j^{[1]}} x_j^{[1]}. \tag{S128}$$

The payoff from layer 2, i.e., $u_i^{[2]} = x_i^{[2]}(r-1)+1$, remains unchanged.

We rewrite Eq. (S128) as

$$\begin{aligned} u_k^{[1]}(\boldsymbol{x}) &= \sum_{\ell=1}^{N} \left(-c \cdot p_{k\ell}^{[1]} \cdot x_k^{[1]} + b \cdot \frac{w_{k\ell}^{[1]}}{s_\ell^{[1]}} \cdot x_\ell^{[1]} \right) \\ &= \sum_{\ell=1}^{N} \left(-c \cdot p_{k\ell} \cdot x_k^{[1]} + b \cdot \frac{w_{\ell k}^{[1]}}{s_\ell^{[1]}} \cdot x_\ell^{[1]} \right) \\ &= \sum_{\ell=1}^{N} \left(-c \cdot p_{k\ell}^{[1]} \cdot x_k^{[1]} + b \cdot p_{\ell k}^{[1]} \cdot x_\ell^{[1]} \right) \\ &= \sum_{\ell=1}^{N} \left(-C_{k\ell} \cdot x_k^{[1]} + B_{k\ell} \cdot x_\ell^{[1]} \right). \end{aligned} \tag{S129}$$

Then, Eq. (S36) becomes

$$\begin{aligned} \frac{\mathrm{d}}{\mathrm{d}\delta} \rho_A^{[1]}(\boldsymbol{\xi}) \bigg|_{\delta=0} = \sum_{i,j,k=1}^{N} \pi_i^{[1]} m_{k;ji}^{[1]} \Bigg\{ - \Bigg[\sum_{\ell=1}^{N} \left(\beta_{jk}^{\boldsymbol{\xi}[1]} - \beta_{ik}^{\boldsymbol{\xi}[1]} \right) C_{k\ell} - \left(\beta_{jl}^{\boldsymbol{\xi}[1]} - \beta_{il}^{\boldsymbol{\xi}[1]} \right) B_{k\ell} \Bigg] \\ + \left[\left(\gamma_{jk}^{\boldsymbol{\xi}[1,2]} - \gamma_{ik}^{\boldsymbol{\xi}[1,2]} \right) (r-1) + \left(\eta_j^{\boldsymbol{\xi}[1]} - \eta_i^{\boldsymbol{\xi}[1]} \right) \right] \Bigg\}. \end{aligned} \tag{S130}$$

Note that the only difference between Eqs. (S36) and (S130) is the use of $B_{k\ell}$ instead of $B_{\ell k}$. Therefore, the condition for the cooperator to be selected in layer 1, i.e.,

$$\frac{\mathrm{d}}{\mathrm{d}\delta} \rho_A^{[1]}(\boldsymbol{\xi}) \bigg|_{\delta=0} > 0, \tag{S131}$$

is given by

$$\begin{aligned} \sum_{i,j,k=1}^{N} \pi_i^{[1]} m_{k;ji}^{[1]} \Bigg\{ - \Bigg[\sum_{\ell=1}^{N} \left(\beta_{jk}^{\boldsymbol{\xi}[1]} - \beta_{ik}^{\boldsymbol{\xi}[1]} \right) C_{k\ell} - \left(\beta_{jl}^{\boldsymbol{\xi}[1]} - \beta_{il}^{\boldsymbol{\xi}[1]} \right) B_{k\ell} \Bigg] \\ + \left[\left(\gamma_{jk}^{\boldsymbol{\xi}[1,2]} - \gamma_{ik}^{\boldsymbol{\xi}[1,2]} \right) (r-1) + \left(\eta_j^{\boldsymbol{\xi}[1]} - \eta_i^{\boldsymbol{\xi}[1]} \right) \right] \Bigg\} > 0. \end{aligned} \tag{S132}$$

By substituting Eq. (S42) in Eq. (S132), we obtain

$$\sum_{i=1}^{N}\pi_i^{[1]}\sum_{\ell=1}^{N}\left[-\beta_{ii}^{\boldsymbol{\xi}[1]}C_{i\ell}+\beta_{i\ell}^{\boldsymbol{\xi}[1]}B_{i\ell}+\gamma_{ii}^{\boldsymbol{\xi}[1,2]}(r-1)+\eta_i^{\boldsymbol{\xi}[1]}\right]>$$
$$\sum_{i,j=1}^{N}\pi_i^{[1]}\left(p^{[1]}\right)_{ij}^{(2)}\sum_{\ell=1}^{N}\left[-\beta_{ij}^{\boldsymbol{\xi}[1]}C_{jl}+\beta_{il}^{\boldsymbol{\xi}[1]}B_{j\ell}+\gamma_{ij}^{\boldsymbol{\xi}[1,2]}(r-1)+\eta_i^{\boldsymbol{\xi}[1]}\right]. \tag{S133}$$

By substituting $C_{kl}=cp_{kl}^{[1]}$ and $B_{lk}=bp_{lk}^{[1]}$ in Eq. (S133), we obtain

$$\sum_{i=1}^{N}\pi_i^{[1]}\sum_{\ell=1}^{N}\left[-\beta_{ii}^{\boldsymbol{\xi}[1]}cp_{i\ell}^{[1]}+\beta_{i\ell}^{\boldsymbol{\xi}[1]}bp_{\ell i}^{[1]}+\gamma_{ii}^{\boldsymbol{\xi}[1,2]}(r-1)+\eta_i^{\boldsymbol{\xi}[1]}\right]>$$
$$\sum_{i,j=1}^{N}\pi_i^{[1]}\left(p^{[1]}\right)_{ij}^{(2)}\sum_{\ell=1}^{N}\left[-\beta_{ij}^{\boldsymbol{\xi}[1]}cp_{j\ell}^{[1]}+\beta_{i\ell}^{\boldsymbol{\xi}[1]}bp_{\ell j}^{[1]}+\gamma_{ij}^{\boldsymbol{\xi}[1,2]}(r-1)+\eta_i^{\boldsymbol{\xi}[1]}\right]. \tag{S134}$$

By substituting Eqs. (S47) and (S48) in Eq. (S134), we obtain

$$b\left\{\sum_{i=1}^{N}\pi_i^{[1]}\sum_{\ell=1}^{N}\beta_{i\ell}^{\boldsymbol{\xi}[1]}p_{\ell i}^{[1]}-\sum_{i,j=1}^{N}\pi_i^{[1]}\left(p^{[1]}\right)_{ij}^{(2)}\sum_{\ell=1}^{N}\beta_{i\ell}^{\boldsymbol{\xi}[1]}p_{\ell j}^{[1]}\right\}+c\theta_2^{\boldsymbol{\xi}[1]}-(r-1)\,\phi_{2,0}^{\boldsymbol{\xi}[1,2]}>0. \tag{S135}$$

Equation (S135) holds true for the game layer (i.e., layer 1) under the dB rule no matter whether the constant-selection layer (i.e., layer 2) obeys the dB or Bd rule. Under the dB-dB rule, the recurrence equations for computing $\beta_{ii}^{\boldsymbol{\xi}[1]}, \beta_{ij}^{\boldsymbol{\xi}[1]}$ (with $i\neq j$), $\gamma_{ij}^{\boldsymbol{\xi}[1,2]}$, and $\eta_i^{\boldsymbol{\xi}[1]}$ are Eqs. (S52), (S54), (S56), and (S57), respectively. Under the dB-Bd rule, the recurrence equations are Eqs. (S52), (S54), (S72), and (S57).

The condition under which the mutant is selected in layer 2, i.e.,

$$\left.\frac{\mathrm{d}}{\mathrm{d}\delta}\rho_A^{[2]}(\boldsymbol{\xi})\right|_{\delta=0}>0, \tag{S136}$$

is given by

$$\sum_{i,j,k=1}^{N}\pi_i^{[2]}m_{k;ji}^{[2]}\left\{-\left[\sum_{\ell=1}^{N}\left(\gamma_{jk}^{\boldsymbol{\xi}[2,1]}-\gamma_{ik}^{\boldsymbol{\xi}[2,1]}\right)C_{k\ell}-\left(\gamma_{jl}^{\boldsymbol{\xi}[2,1]}-\gamma_{il}^{\boldsymbol{\xi}[2,1]}\right)B_{k\ell}\right]\right.$$
$$\left.+\left[\left(\beta_{jk}^{\boldsymbol{\xi}[2]}-\beta_{ik}^{\boldsymbol{\xi}[2]}\right)(r-1)+\left(\eta_j^{\boldsymbol{\xi}[1]}-\eta_i^{\boldsymbol{\xi}[1]}\right)\right]\right\}>0. \tag{S137}$$

For the dB-dB updating rule, by substituting Eqs. (S47) and (S48) in Eq. (S137), we obtain

$$\sum_{i=1}^{N}\pi_i^{[2]}\sum_{\ell=1}^{N}\left[-\gamma_{ii}^{\boldsymbol{\xi}[2,1]}cp_{i\ell}^{[1]}+\gamma_{i\ell}^{\boldsymbol{\xi}[2,1]}bp_{\ell i}^{[1]}+\beta_{ii}^{\boldsymbol{\xi}[2]}(r-1)+\eta_i^{\boldsymbol{\xi}[2]}\right]>$$
$$\sum_{i,j=1}^{N}\pi_i^{[2]}\left(p^{[2]}\right)_{ij}^{(2)}\sum_{\ell=1}^{N}\left[-\gamma_{ij}^{\boldsymbol{\xi}[2,1]}cp_{j\ell}^{[1]}+\gamma_{i\ell}^{\boldsymbol{\xi}[2,1]}bp_{\ell j}^{[1]}+\beta_{ij}^{\boldsymbol{\xi}[2]}(r-1)+\eta_i^{\boldsymbol{\xi}[2]}\right]. \tag{S138}$$

For the dB-Bd updating rule, we similarly obtain

$$\sum_{i,j=1}^{N}\pi_i^{[2]}p_{ji}^{[2]}\sum_{\ell=1}^{N}\left[-\gamma_{ii}^{\boldsymbol{\xi}[2,1]}cp_{i\ell}^{[1]}+\gamma_{i\ell}^{\boldsymbol{\xi}[2,1]}bp_{i\ell}^{[1]}+\beta_{ii}^{\boldsymbol{\xi}[2]}(r-1)+\eta_i^{\boldsymbol{\xi}[2]}\right]>$$
$$\sum_{i,j=1}^{N}\pi_i^{[2]}p_{ji}^{[2]}\sum_{\ell=1}^{N}\left[-\gamma_{ij}^{\boldsymbol{\xi}[2,1]}cp_{j\ell}^{[1]}+\gamma_{i\ell}^{\boldsymbol{\xi}[2,1]}bp_{j\ell}^{[1]}+\beta_{ij}^{\boldsymbol{\xi}[2]}(r-1)+\eta_i^{\boldsymbol{\xi}[2]}\right]. \tag{S139}$$

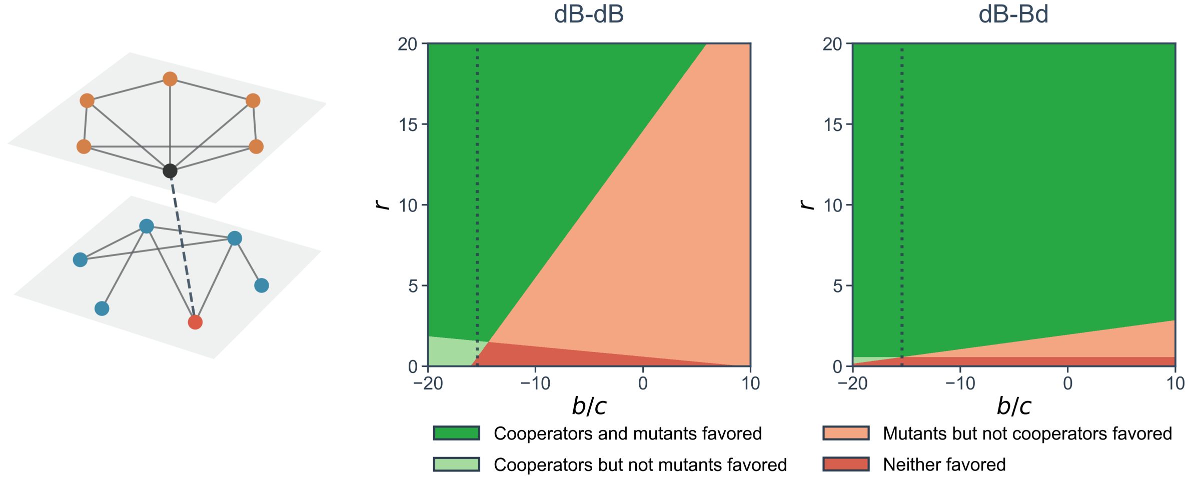

Figure S4: Parameter regions in which the cooperator or mutant is favored under the ff good scheme for the two-layer network used in Fig. 4(b) in the main text (replicated in the left panel of this figure). The vertical dotted lines indicate the $(b/c)^*$ value ($= -15.41$) when the two layers are uncoupled.

Under the dB-dB rule, the recurrence equations for computing $\beta_{ii}^{\boldsymbol{\xi}[1]}$, $\beta_{ij}^{\boldsymbol{\xi}[1]}$ (with $i \neq j$), $\gamma_{ij}^{\boldsymbol{\xi}[1,2]}$, and $\eta_i^{\boldsymbol{\xi}[1]}$ are Eqs. (S52), (S54), (S56), and (S57), respectively. Under the dB-Bd rule, the recurrence equations are Eqs. (S70), (S70), (S72), and (S57).

S9.B Numerical results

In Fig. S4, we show the $(b/c)^*$ and r^* values for the two-layer network used in Fig. 4(b) in the main text, but under the ff goods scheme. For the other three networks used in Fig. 4, the $(b/c)^*$ value is the same between the ff and pf goods because the game layer (i.e., layer 1) in these two-layer networks are regular graphs; Eqs. (S127) and (S128) are identical for regular graphs. For the network shown in Fig. S4, we obtain qualitatively the same behavior of $(b/c)^*$ as a function of r between the two goods schemes; compare Fig. S4(b) and Fig. 4(b). The r^* value as a function of b/c is also similar between the ff and pf goods.

S9.C Random graphs

For the ff goods scheme, we analyzed the condition under which the cooperator and mutant are selected in two-layer ER and BA networks on 15 individuals.

We show in Fig. S5(a) and (b) the results for the two-layer ER and BA networks, respectively, under the dB-dB updating rule. We find that all the layer-1 networks enable cooperation, not spite (see the upper part of Fig. S5(a) and (b)). The responsiveness of $(b/c)^*$ as the constant selection changes, quantified by $|\mathrm{d}(b/c)^*/\mathrm{d}r|$, increases as the edge density in layer 1 increases or that in layer 2 decreases (see the lower part of Fig. S5(a) and (b)). These results are similar to those for the pf goods shown in Fig. 5(a) and (b) in the main text. The results are qualitatively the same for the dB-Bd rule, with overall larger $|\mathrm{d}(b/c)^*/\mathrm{d}r|$ than the case of the dB-dB rule (see Fig. S5(c) and (d)). Finally, we have verified that the results do not substantially change when we switch the measurement of $|\mathrm{d}(b/c)^*/\mathrm{d}r|$ from the median and percentiles to the mean and standard deviation (see Fig. S6).

S9.D Empirical networks

We show in Fig. S7 the results for the VC7 and LLF law two-layer networks under the ff goods scheme. The results are qualitatively the same as those under the pf goods scheme. It should be noted that the

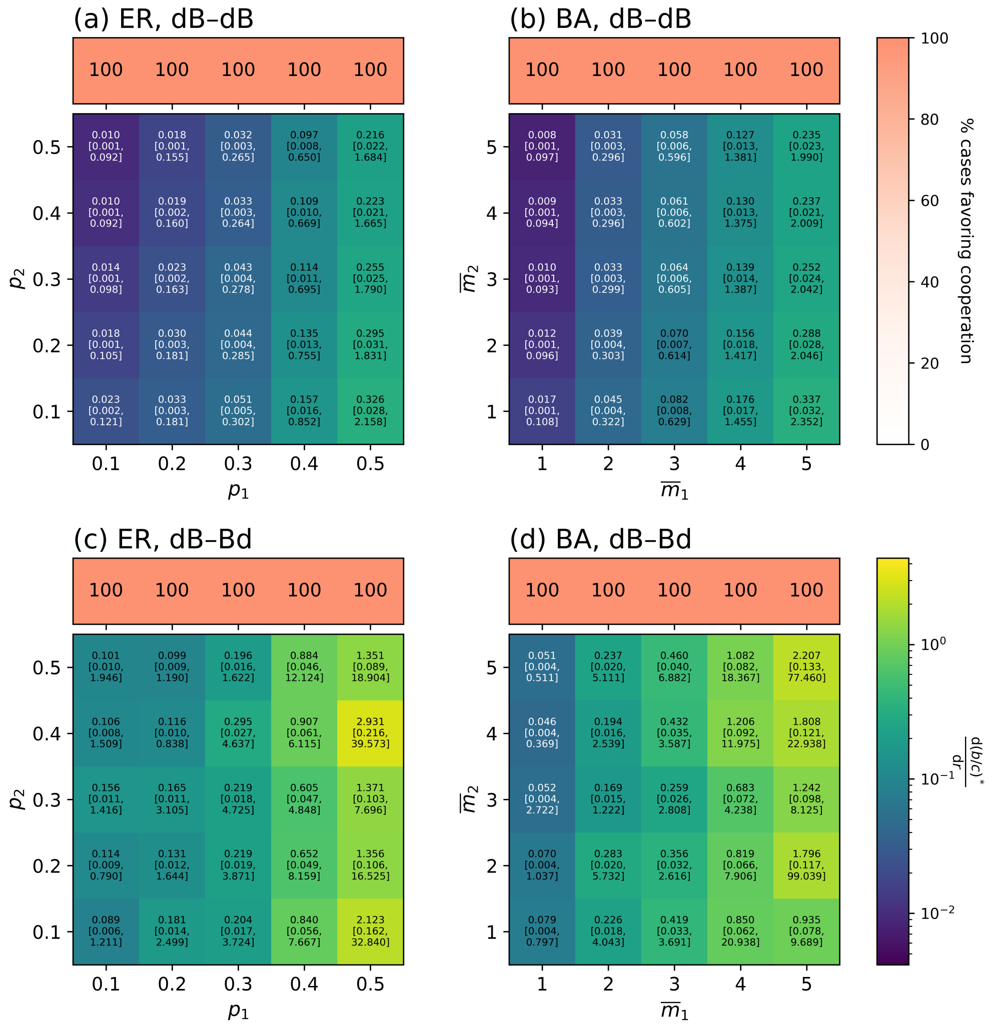

Figure S5: Evolution of cooperation in two-layer ER and BA networks under the ff goods scheme. (a) ER, dB-dB rule. (b) BA, dB-dB rule. (c) ER, dB-Bd rule. (d) BA, dB-Bd rule. The upper part of each panel shows the fraction of pairs of single-layer network and initial condition that yield cooperation when $b/c > (b/c)^*$ for a threshold value $(b/c)^* > 0$. The fraction values are the same between (a) and (c) and between (b) and (d) because whether or not $(b/c)^* > 0$ in layer 1 does not depend on the updating rule used in layer 2. The lower part of each panel shows the median along with the 5th and 95th percentiles (in square brackets) of $|\mathrm{d}(b/c)^*/\mathrm{d}r|$ for pairs of two-layer network and initial condition. Each two-layer network is composed of $N = 15$ individuals.

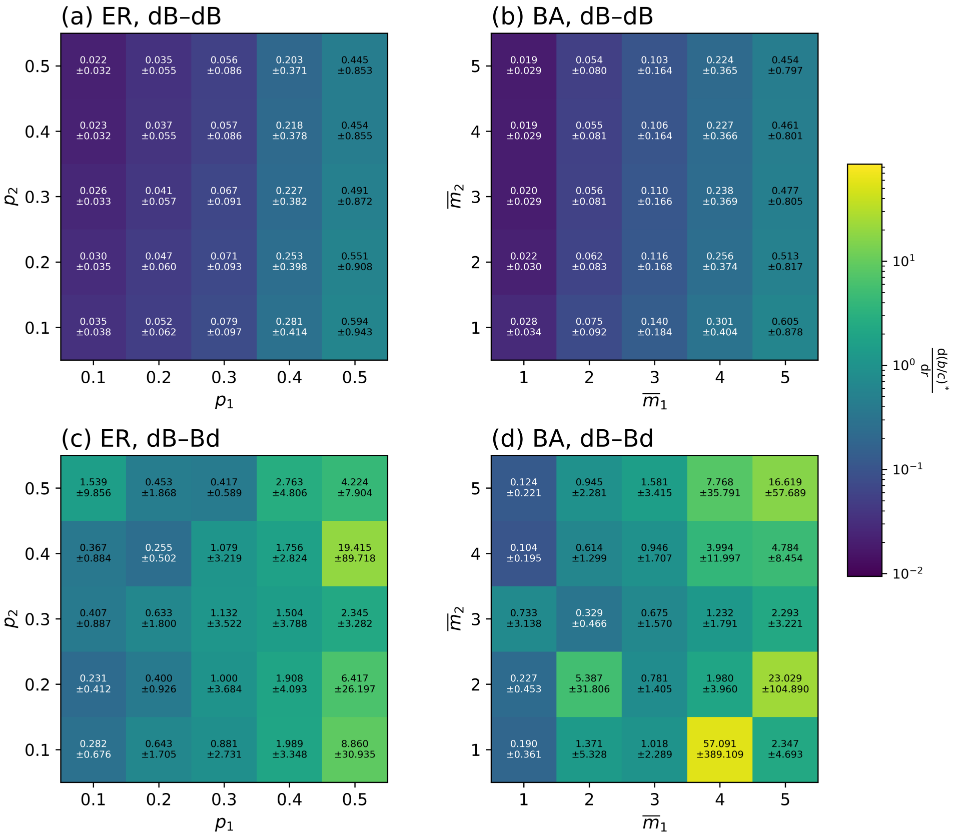

Figure S6: Mean, μ, and standard deviation, σ, of $|\mathrm{d}(b/c)^*/\mathrm{d}r|$ for two-layer ER and BA networks with $N = 15$ individuals under the ff goods scheme. (a) ER, dB-dB rule. (b) BA, dB-dB rule. (c) ER, dB-Bd rule. (d) BA, dB-Bd rule. See the caption of Fig. S3 for details of the computation.

quantitative difference between the ff and pf goods schemes is large in this case; for the VC7 network, we have obtained $(b/c)^* = -298.7$, which is far from $(b/c)^* = -89.5$ in the case of the pf goods shown in the main text; for the LLF law network, we have obtained $(b/c)^* = 28.66$ under the ff goods scheme, whereas the main text shows $(b/c)^* = 52.4$ under the pf goods scheme.

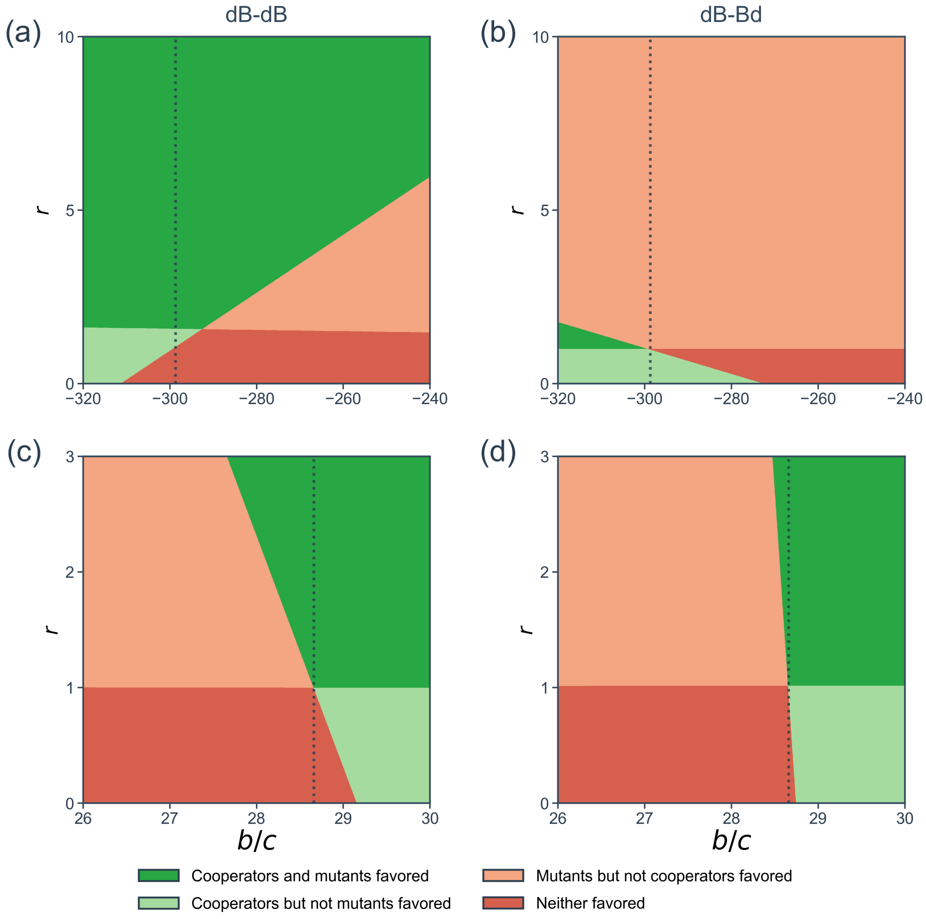

Figure S7: Parameter regions in which the cooperator or mutant is selected in empirical two-layer networks under the ff goods scheme. The vertical lines represent the $(b/c)^*$ value for the uncoupled layer-1 network. (a) VG7, dB-dB. (b) VG7, dB-Bd. (c) LLF, dB-dB. (d) LLF, dB-Bd. The single-layer VG7 and LLF networks yield $(b/c)^* = -298.7$ and $(b/c)^* = 28.66$, respectively.

S10 Fixation time

We computed the fixation time for cooperation under the dB-dB rule combined with the pf good scheme. We used the four synthetic two-layer networks used in Fig. 4 and the VC7 network. We did not use the LLF network because numerical estimation of the fixation time of cooperation when starting from a single cooperator is computationally demanding. We generated 1,600,000 runs that finished with fixation of cooperation in layer 1 no matter whether the resident or mutant type fixated in layer 2 for the four synthetic networks. We reduced the number of runs to 800,000 for the VC7 network. Then, we recorded the time to fixation of cooperation and averaged it over all the runs. It should be noted that we defined the fixation time as the time at which the cooperator has fixated regardless of whether or not fixation has been attained in layer 2. This choice is to enable fair comparison between the two-layer and one-layer networks; if we wait for the fixation of both layers, then the fixation time would be obviously shorter for one-layer networks than the two-layer networks whose layer-1 network is the same as the one-layer network under comparison. As reference, we also computed the mean fixation time for the one-layer network that is layer 1 of each two-layer network analyzed.

We show the mean fixation time for cooperation in Fig. S8. We find that the fixation time for the four synthetic two-layer networks is not much larger than that for the corresponding one-layer networks across the parameter values and the choice of two-layer network. For the VC7 network, the two-layer network needs about 1.6–2.8 more time to fixation than one-layer counterparts.

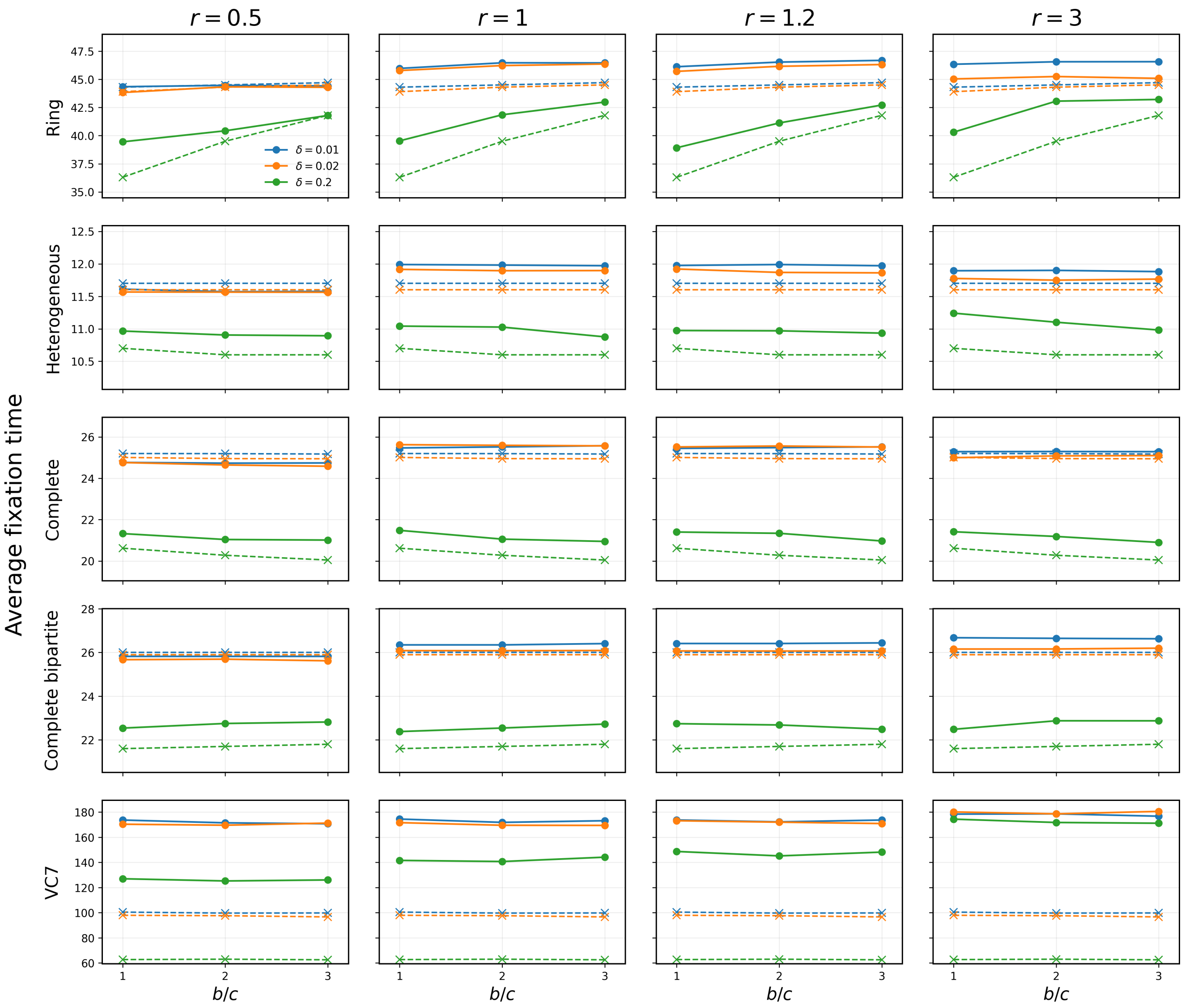

Figure S8: Mean fixation time for cooperation in the four synthetic networks and the VC7 network. The ring, heterogeneous, complete, and complete bipartite networks refer to the two-layer networks used in Fig. 4(a), (b), (c), and (d), respectively. The solid and dashed lines represent the mean fixation time of cooperation in layer 1 of the two-layer networks and the single-layer counterparts, respectively. We used $r \in \{0.5, 1, 1.2, 3\}$.

Supplementary References

[1] Q. Su, A. McAvoy, Y. Mori, and J. B. Plotkin, Evolution of prosocial behaviours in multilayer populations, *Nature Human Behaviour*, 6, 338–348, 2022.

[2] B. Allen and A. McAvoy, A mathematical formalism for natural selection with arbitrary spatial and genetic structure, *Journal of Mathematical Biology*, 78, 1147–1210, 2019.

[3] A. McAvoy and B. Allen, Fixation probabilities in evolutionary dynamics under weak selection, *Journal of Mathematical Biology*, 82, 14, 2021.

[4] R. A. Fisher, *The Genetical Theory of Natural Selection: A Complete Variorum Edition.* Oxford University Press, Oxford, UK, 1999.

[5] P. D. Taylor, Allele-frequency change in a class-structured population, *American Naturalist*, 135, 95–106, 1990.

[6] P. D. Taylor, Inclusive fitness arguments in genetic models of behaviour, *Journal of Mathematical Biology*, 34, 654–674, 1996.

[7] A. Grafen, A theory of Fisher's reproductive value, *Journal of Mathematical Biology*, 53, 15–60, 2006.

[8] W. Maciejewski, Reproductive value in graph-structured populations, *Journal of Theoretical Biology*, 340, 285–293, 2014.

[9] A. McAvoy, B. Allen, and M. A. Nowak, Social goods dilemmas in heterogeneous societies, *Nature Human Behaviour*, 4, 819–831, 2020.

[10] V. Sood, T. Antal, and S. Redner, Voter models on heterogeneous networks, *Physical Review E*, 77, 041121, 2008.

[11] N. Masuda and H. Ohtsuki, Evolutionary dynamics and fixation probabilities in directed networks, *New Journal of Physics*, 11, 033012, 2009.
